# Smart Imitator: Learning from Imperfect Clinical Decisions

**DOI:** 10.1093/jamia/ocae320

**Published:** 2025-01-10

**Authors:** Dilruk Perera, Siqi Liu, Kay Choong See, Mengling Feng

**Affiliations:** Institute of Data Science, National University of Singapore, 117602, Singapore; Saw Swee Hock School of Public Health, National University of Singapore, 117549, Singapore; Institute of Data Science, National University of Singapore, 117602, Singapore; NUS Graduate School—ISEP, 119077, Singapore; Yong Loo Lin School of Medicine, National University of Singapore, 117597, Singapore; Institute of Data Science, National University of Singapore, 117602, Singapore; Saw Swee Hock School of Public Health, National University of Singapore, 117549, Singapore

**Keywords:** health care AI, clinical decision-making, reinforcement learning (RL), adversarial imitation learning, imitation learning (IL)

## Abstract

**Objectives:**

This study introduces Smart Imitator (SI), a 2-phase reinforcement learning (RL) solution enhancing personalized treatment policies in healthcare, addressing challenges from imperfect clinician data and complex environments.

**Materials and Methods:**

Smart Imitator’s first phase uses adversarial cooperative imitation learning with a novel sample selection schema to categorize clinician policies from optimal to nonoptimal. The second phase creates a parameterized reward function to guide the learning of superior treatment policies through RL. Smart Imitator’s effectiveness was validated on 2 datasets: a sepsis dataset with 19 711 patient trajectories and a diabetes dataset with 7234 trajectories.

**Results:**

Extensive quantitative and qualitative experiments showed that SI significantly outperformed state-of-the-art baselines in both datasets. For sepsis, SI reduced estimated mortality rates by 19.6% compared to the best baseline. For diabetes, SI reduced HbA1c-High rates by 12.2%. The learned policies aligned closely with successful clinical decisions and deviated strategically when necessary. These deviations aligned with recent clinical findings, suggesting improved outcomes.

**Discussion:**

Smart Imitator advances RL applications by addressing challenges such as imperfect data and environmental complexities, demonstrating effectiveness within the tested conditions of sepsis and diabetes. Further validation across diverse conditions and exploration of additional RL algorithms are needed to enhance precision and generalizability.

**Conclusion:**

This study shows potential in advancing personalized healthcare learning from clinician behaviors to improve treatment outcomes. Its methodology offers a robust approach for adaptive, personalized strategies in various complex and uncertain environments.

## Introduction

Complexity and uncertainty in medical treatments, driven by diverse patient responses and intricate disease mechanisms, highlight limitations of traditional one-size-fits-all approaches.^1^ These methods fail to account for individual patient differences, leading to suboptimal outcomes and potential adverse effects. Thus, there is a critical need for advanced, data-driven decision support systems to help clinicians make informed, real-time decisions. The goal is to develop intelligent agents that learn and adapt treatment policies to the nuances of clinical practices, accommodating diverse patients.

Reinforcement learning (RL) is considered a promising approach for this task, as it enables agents to navigate complex conditions independently, potentially identifying effective strategies without relying on predefined, optimal plans.^1^^,^^2^ Through trial and error, RL agents have the potential to identify highly effective interventions that may improve patient outcomes.^2–5^ While RL provides a powerful framework for learning optimal strategies in complex and dynamic environments, its application in healthcare is still emerging, with growing evidence suggesting significant potential benefits.^1^^,^^6^ However, traditional RL solutions in healthcare face significant limitations due to the complexity and variability in medical conditions.^6^^,^^7^

### Clinician-derived data limitations

Traditional RL applications like gaming^8^ and robotics^9^ learn through active engagement in simulated environments. However, in healthcare, ethical, privacy, and practical constraints limit RL learning to primarily rely on historical clinician-patient interactions.^7^ While prospective randomized trials, such as Sequential Multiple Assignment Randomized Trials (SMARTs), provide an alternative for RL systems to dynamically learn treatment policies, their high cost, complexity, and ethical concerns make them difficult to implement in healthcare.^10^^,^^11^ Consequently, RL applications in healthcare are predominantly trained on retrospective observational data.^3^^,^^6^ Moreover, significant variability in clinical practice, influenced by differing expertise and institutional guidelines, complicates data consistency, posing challenges for RL systems in developing effective treatment policies.^7^^,^^12^ The variability and potential misalignment of clinician actions with optimal care pathways can misguide RL agents, resulting in ineffective or potentially harmful treatment policies.^13^^,^^14^

To mitigate selection bias and variability, traditional methods such as statistical adjustment techniques—for example, propensity score matching^15^ and inverse probability weighting^16^—have been used. These techniques aim to balance datasets by accounting for confounding variables, reducing bias, and improving representativeness. Additionally, standardization efforts, including developing clinical guidelines and protocols^17^ also aim to minimize variability in treatment practices. However, these approaches may not fully address the complexities inherent in clinician-derived data. Statistical adjustments often struggle to capture all confounding factors, especially in high-dimensional healthcare data, and can be sensitive to model specifications.^18^ Furthermore, standardizing clinical practices is challenging due to the dynamic nature of medical knowledge and varying resources across institutions.^19^

### Environmental complexity and uncertainty

High-dimensional data and complex patient trajectories lead to slow or even nonconvergence in RL models.^5^^,^^12^^,^^20^ Chronic conditions such as diabetes require long-term treatments, resulting in sparse rewards and complicating the evaluation of treatment effectiveness. Conversely, acute conditions such as sepsis involve rapidly changing clinical settings that obscure immediate intervention effects, adding complexity to the training process. These factors challenge the attribution of patient outcomes to specific clinician actions (ie, credit assignment problem). Heterogeneity among patients complicates generalizations across populations, increasing uncertainty of the task. Relying on infrequent and delayed outcomes such as mortality further complicates the process. Although intermittent rewards have been proposed to mitigate some issues,^21^ they require extensive expert input and often lack generalizability,^22^ highlighting the need for robust RL solutions to navigate healthcare complexities and uncertainties.

Despite recent RL advancements like imitation learning (IL) and inverse reinforcement learning (IRL)^23^ to mitigate these challenges and directly learn from clinician behavior without predefined rewards, significant gaps remain. Early IL approaches mimicked all clinician actions, assuming optimality. Later methods focused only on the actions leading to successful outcomes, still assuming optimality. However, these assumptions fail in complex healthcare environments where observational data are imperfect and even the trajectories leading to successful outcomes can include suboptimal decisions, and vice versa introducing ambiguities and misclassifications in treatment policies.^24–27^ Inverse reinforcement learning aimed to infer the underline reward function that clinicians optimize, but is hindered by the high-dimensional, noisy medical data.^28^^,^^29^ Additionally, IRL also assumes near-optimality in observational data, leading to nonrobust, nongeneralizable reward functions.^30^^,^^31^ Additionally, these methods are limited by the quality and coverage of demonstrator actions.

To address these limitations, we introduce the Smart Imitator (SI), a novel learning framework enhancing RL solutions through a structured, 2-phase learning process. Phase 1 introduces adversarial cooperative imitation learning (ACIL) to categorize and rank clinician actions into a structured hierarchy of policies. This allows for a nuanced analysis of decision-making processes and mitigates the impact of suboptimal or inconsistent clinical actions. Phase 2 employs an innovative IRL technique to approximate the underlying reward function, improving the precision in evaluating and prioritizing clinical actions. By leveraging this approach, our framework effectively mitigates selection bias and accounts for variability in clinical practice, leading to more robust and generalizable treatment policies that can potentially surpass the observational data used to train the model. The SI framework aims to address key challenges in healthcare RL applications by enhancing treatment policy accuracy and potentially reducing the risks associated with generic treatment practices. Although motivated and tested within healthcare, the introduced 2-phase learning process is generic and can be applied beyond healthcare to surpass expert policies under complex and uncertain environments.

Our main contributions are as follows:

- We introduce a novel 2-phase RL framework that integrates ACIL with inverse RL. This innovative approach significantly enhances policy accuracy and adaptability across diverse RL applications, including but not limited to healthcare.
- We develop a clinically guided nonoptimal sample selection schema to identify and utilize suboptimal expert observational data effectively, assisting the ranking of clinician policies.
- We validate the SI framework through comprehensive case studies on sepsis and diabetes treatment, demonstrating its effectiveness compared to existing solutions and its potential to improve treatment efficacy and survival rates.

## Literature review

### Dynamic treatment regimes and statistical approaches

Dynamic treatment regimes (DTRs) provide a robust framework for sequential, personalized treatment decisions based on patient responses over time. Traditionally, statistical methods like A-learning have optimized DTRs using both clinical trial and observational data.^32–34^ Additionally, Q-learning, originating from RL, has been adapted for DTRs to derive adaptive decision rules by analyzing patient trajectories.^35^^,^^36^ These methods have considerably advanced personalized medicine, particularly in fields where individual variability significantly impacts treatment effectiveness.^35^^,^^37^

However, these statistical methods often rely on strong assumptions about the data-generating process and may struggle with the high-dimensional, complex data with high variability and imperfections typical in real-world settings.^6^^,^^7^ Our work builds on these foundations by incorporating RL techniques to handle such complexities more effectively.

### RL in health care and challenges

Reinforcement learning has demonstrated significant success in managing complex sequential tasks, such as game playing,^8^ autonomous driving, and robotics.^38^ Leveraging these advances, RL has been applied in healthcare to assist clinicians in disease diagnosis,^3^ and treatment recommendations,^39^^,^^40^ often by processing demographic and clinical data from electronic health records (EHRs).^1^ Despite limited clinical implementation, RL has a significant potential in treating complex conditions such as sepsis and diabetes, where standardized treatments are absent.^41–43^ These studies primarily used patient mortality as the reward, which, while critically important, introduce significant challenges. In sepsis, rapid disease progression in intensive care results in delayed rewards for treatment decisions. Conversely, diabetes management involves long-term interventions, creating highly sparse rewards. This exclusive reliance on mortality as a reward restricts RL’s ability to effectively explore and learn.^43^ Recent research has introduced hand-crafted, intermittent rewards to improve treatment exploration.^1^^,^^21^ However, this approach requires expert-driven reward design, which is particularly challenging for complex diseases without standardized treatments. Success is critically dependent on the reward design, which can often lead to unintended outcomes.^41^

### Advancements through imitation learning

Imitation learning (IL) offers an alternative to hand-crafted reward functions by using past interactions to demonstrate desired behavior and guide the agent with certainty.^44^ Imitation learning is widely adopted in fields such as humanoid robotics,^45^ human-computer interaction,^46^ autonomous driving^47^ and gaming.^48^ However, its applications in healthcare are limited since most IL algorithms require online training with interactive feedback, such as policy coaching^49^ or online apprenticeship learning.^50^ Behavior Cloning (BC)^51^ uses supervised learning to predict expert actions, while generative adversarial imitation learning (GAIL),^52^ leveraging Generative Adversarial Networks (GANs),^53^ generates data that mimics expert demonstrations. Both BC and GAIL work with retrospective data but assume expert demonstrations are optimal, which is often not true in healthcare. Policy optimization from demonstration (POfD) relaxes this assumption, allowing agents to explore unseen scenarios.^54^ However, adhering strictly to expert encountered demonstrations can perpetuate errors if demonstrations are imperfect. Accordingly, a recent appraoch named generative adversarial imitation learning with imperfect demonstration and confidence (IC-GAIL) addresses this by emulating only highly rated expert actions,^55^ yet it is impractical in healthcare due to the difficulty of designing an effective oracle. Therefore, training agents to learn from imperfect demonstrations remains crucial, especially in complex treatment environments.

### Adversarial cooperative imitation learning for enhanced learning

Recent advancements in GAIL have employed ACIL to train agents to mimic patient trajectories.^24^^,^^56^ While these studies form the closest baselines to the proposed solution, they suffer from several key limitations.

These approaches tend to rely on the assumption that all actions in surviving trajectories are beneficial and those in deceased trajectories are detrimental. This simplification often fails in complex healthcare settings, where the effectiveness of individual treatment actions can vary significantly, irrespective of the outcome. As a result, this can lead to learning ambiguities, where similar treatments might be classified as both optimal and nonoptimal based solely on final patient outcomes, which complicates the learning process and makes it harder to learn at the granular step-by-step level. Furthermore, strict reliance on observational data restricts the agent’s ability to learn optimal policies beyond the provided samples, making its performance highly dependent on the quality and scope of the data. Moreover, these approaches are typically model-based, relying on predictions of future patient states, which are often error-prone and difficult to model accurately in complex healthcare environments.^20^

In contrast, the proposed SI framework employs a model-free strategy, learning directly from clinician-derived data, avoiding the inaccuracies of patient state transition models. Furthermore, we implement a novel step-level nonoptimal sample selection criteria that categorizes actions into optimal, suboptimal, and nonoptimal groups, resolving ambiguities at the state-action level rather than focusing on trajectory level, based on outcomes. Behavior Cloning is then used to learn policies under the extracted categories to prevent over-reliance on limited observational data. Finally in phase 2, IRL approximates reward functions, enabling agent to explore beyond observed data, offering broader adaptability and precision in clinical settings. These refinements address the limitations of prior models, considerably enhance the performance in complex clinical scenarios.

## Methodology

For a detailed explanation of the foundational techniques such as BC, IRL, and GAIL that underpin our approach, please refer to the Preliminaries section in the Appendix S1.

### Phase 1: policy ranking in imperfect observational data

In phase 1, we learn and categorize clinician-derived policies into 3 categories: optimal $\left(\Pi_{\mathrm{op}}={\left\{\pi_{\mathrm{op},i}\right\}}_{i=1}^{n_{\mathrm{op}}}\right)$, suboptimal $\left(\Pi_{\mathrm{so}}={\left\{\pi_{\mathrm{so},i}\right\}}_{i=1}^{n_{\mathrm{so}}}\right)$, and nonoptimal $\left(\Pi_{\mathrm{no}}={\left\{\pi_{\mathrm{no},i}\right\}}_{i=1}^{n_{\mathrm{no}}}\right)$, where $n_{\mathrm{op}}$, $n_{\mathrm{so}}$, and $n_{\mathrm{no}}$ represent the number of policies within each category.

#### Nonoptimal policy $\left(\Pi_{\mathrm{no}}\right)$

We learn $\Pi_{\mathrm{no}}$ using clear nonoptimal, observational data that lead to immediate adverse effects, especially when alternative more effective intervention options were evident. To identify these samples, we introduce a clinically guided one-step nonoptimal sample selection schema as an indicator function to evaluate state-action pairs $\left(s,\;a\right)∼D_{e}$ from a mixture of optimal and nonoptimal observational data $D_{e}$ (see “Nonoptimal Sample Selection Schemas” for more details on the schema). This schema mimics an oracle, identifying nonoptimal samples without manual labels or biased parameterized models. It specifically assesses the immediate impact of action $a_{i}$ on state $s_{i}$, streamlining the assessment within complex and extended patient trajectories. Note that establishing nonoptimal criteria is more feasible and less error-prone than establishing strict optimality criteria from limited and imperfect observational data.

Using the schema-selected nonoptimal samples (${\tilde{D}}_{\mathrm{op}}$), we obtain $\Pi_{\mathrm{no}}$ by defining a BC objective^57^ that specifically imitates nonoptimal behavior as follows:


LΠno=Es, a∼D∼op-log π⁡a|s.

Inspired by this approach, one may argue that final treatment policy could be learned by directly avoiding nonoptimal samples using a standard BC approach. However, this method is fundamentally flawed for 2 reasons. First, it inefficiently utilizes available observational data, heavily relying on the limited diversity and coverage of selected samples, which may result in a policy with poor generalizability and compounding errors.^58^^,^^59^ Second, constructing an optimal policy by solely avoiding nonoptimal samples places an excessive burden on the selection schema to accurately discern true optimality. Introducing suboptimal categories and ranking helps to alleviate these issues.

#### Optimal demonstrator policy $\left(\Pi_{\mathrm{op}}\right)$

We extract $\Pi_{\mathrm{op}}$ without direct expert feedback or external rewards using 2 datasets: ${\tilde{D}}_{no}$, identified through nonoptimal sample selection schema, and the remaining set ${\tilde{D}}_{\mathrm{op}}=D_{e}-{\tilde{D}}_{\mathrm{no}}$. Since the schema isolates clear nonoptimal samples, ${\tilde{D}}_{\mathrm{op}}$ may not include absolute optimal samples. However, we use ${\tilde{D}}_{\mathrm{op}}$ to obtain demonstrator optimal policy, laying the foundation for a more optimal policy in subsequent stages.

**Figure 1.**
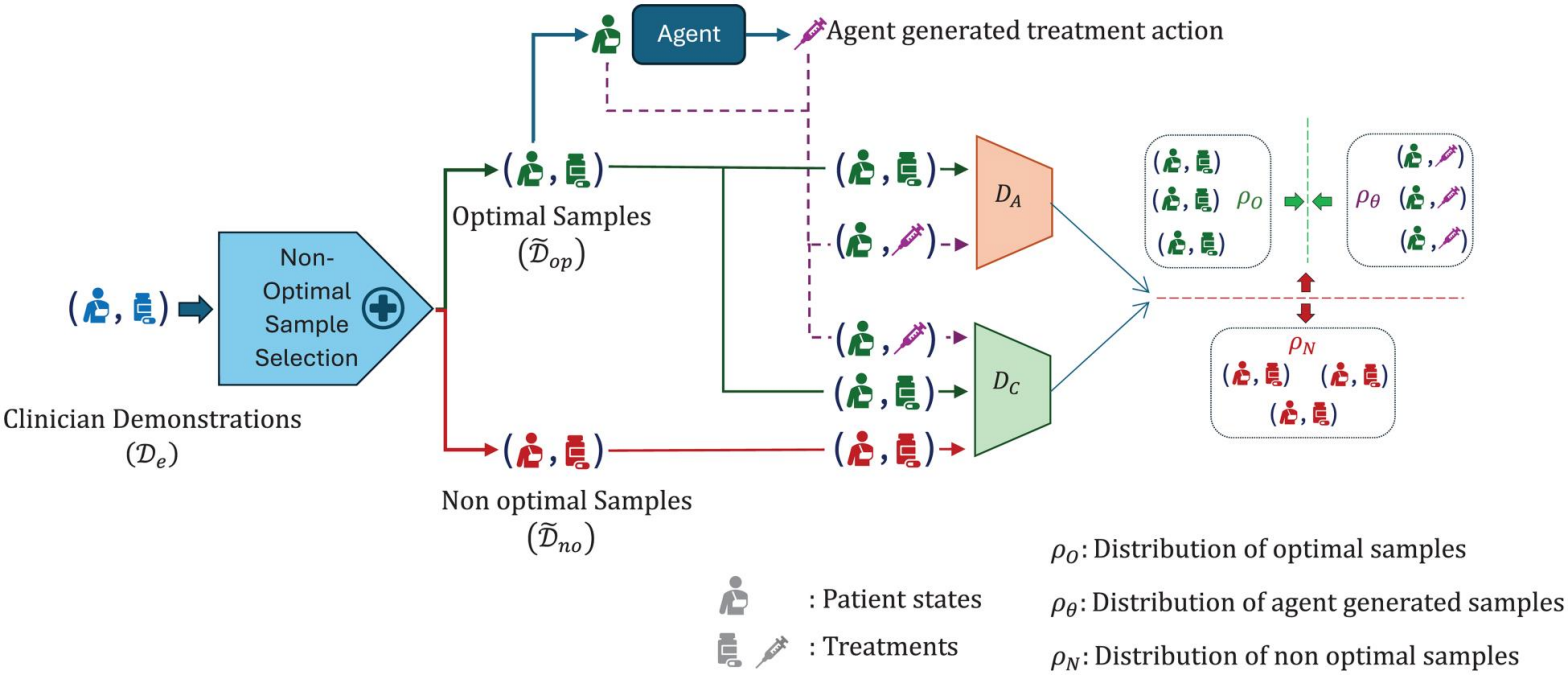
Optimal policy learning process in phase 1.

We introduce an ACIL approach, employing 2 discriminators, an adversarial discriminator ($D_{A}$) and a cooperative discriminator ($D_{C}$), to train an RL agent to effectively recommend optimal treatments (see Figure 1). This approach involves a strategic 3-player min-max game. The agent generates state-action pairs (s, a) using $\Pi_{\mathrm{op}}$. $D_{A}$ evaluates the policy’s accuracy in replicating samples from ${\tilde{D}}_{\mathrm{op}}$, while $D_{C}$ assists the agent by evaluating the authenticity and optimality of generated pairs, ensuring they mirror the quality of $D_{\mathrm{op}}$. The dual-discriminator approach creates a dynamic training environment, enhancing the agent’s adaptability and ensuring the learned policy aligns with high-quality treatments.

*Policy update*: $\Pi_{\mathrm{op}}\left(\phi \right)$ is updated to align with optimal treatments, ensuring generated actions are classified as optimal by both discriminators.


$$L\left(\Pi_{\mathrm{op}}\left(\phi \right)\right)=\underset{\phi }{\min}\left[\lambda_{A}L_{A}-\lambda_{C}L_{C}\right],$$

where $\lambda_{A}$ and $\lambda_{C}$ balance adversarial and cooperative losses ($L_{A}$ and $L_{C}$).


$$\begin{array}{l} L_{A}=E_{\left(s,\;a\right)∼\Pi_{\mathrm{op}}\left(\phi \right)}\;\left[\log\left(1-D_{A}\left(s,a\right)\right)\right]+\;E_{\left(s,\;a\right)∼{\tilde{D}}_{\mathrm{op}}}\;\left[\log D_{A}\left(s,a\right)\right],\\ L_{C}=E_{\left(s,\;a\right)∼\Pi_{\mathrm{op}}\left(\phi \right)}\;\left[\log D_{C}\left(s,a\right)\right]\;+\;E_{\left(s,\;a\right)∼{\tilde{D}}_{\mathrm{no}}}\;\left[\log\left(1-D_{C}\left(s,a\right)\right)\right], \end{array}$$

where $D_{A}\left(s,a\right)$ and $D_{C}\left(s,a\right)$ are corresponding discriminator’s assessment of action a on patient state s.

*Adversarial discriminator (* $D_{A}$ *) update*: Parameterized by $\omega_{A}$, $D_{A}:S\times A\to \left[0,1\right]$ differentiates between the actions generated by $\Pi_{\mathrm{op}}\left(\phi \right)$ and those from ${\tilde{D}}_{\mathrm{op}}$, aiming to accurately identify the origin of each action.


$$\underset{\omega_{A}}{\max}\;\left[E_{\left(s,\;a\right)∼{\tilde{D}}_{\mathrm{op}}}\left[\log D_{A}\left(s,a\right)\right]+E_{\left(s,\;a\right)∼\Pi_{\mathrm{op}}\left(\phi \right)}\left[\log\left(1-D_{A}\left(s,a\right)\right)\right]\right].$$

*Cooperative discriminator (* $D_{C}$ *) update:* Parameterized by $\omega_{C}$, $D_{C}:S\times A\to \left[0,1\right]$ ensures that actions generated by $\Pi_{\mathrm{op}}\left(\phi \right)\;$align with high-quality demonstrator actions and deviate from nonoptimal samples.


$$\underset{\omega_{C}}{\max}\;\left[E_{\left(s,\;a\right)∼{\tilde{D}}_{\mathrm{op}}\;\cup {\;\Pi }_{\mathrm{op}}\left(\phi \right)}\left[\log D_{C}\left(s,a\right)\right]+E_{\left(s,\;a\right)∼{\tilde{D}}_{\mathrm{no}}}\left[\log\left(1-D_{C}\left(s,a\right)\right)\right]\right]\;.$$

This comprehensive training approach enables the agent to refine its strategy continuously through interaction and feedback from both discriminators. The detailed discriminator updates ensure the policy not only replicates but also potentially exceeds the decisions demonstrated in clinical practice by effectively learning from optimal and suboptimal samples.

#### Suboptimal policy $\left(\Pi_{\mathrm{so}}\right)$

We develop a suboptimal dataset ${\tilde{D}}_{\mathrm{so}}$ by blending actions from learned $\Pi_{\mathrm{op}}$ and $\Pi_{\mathrm{no}}$ policies:


D∼so={(s,a)|∀(s,aop)∈Πop, ∀(s,ano)∈Πno, a= {aop with probability α ano with probability (1-α)},

where blend ratio $\alpha \in \left[0,1\right]$ controls the mix of optimal and suboptimal samples, creating a range of clinical scenarios. We derive $\Pi_{\mathrm{so}}$ using BC to maximize the likelihood of actions in ${\tilde{D}}_{\mathrm{so}}$:


$$\Pi_{\mathrm{so}}=\underset{\pi }{\arg\max}\sum_{\left(s,a\right)\in {\tilde{D}}_{\mathrm{so}}}\log\pi \left(a|s\right).$$

This produces a suboptimal policy that balances theoretical ideals with practical clinical scenarios.

Adjusting α simulates suboptimal policies under varying levels of clinical decision variability. In complex conditions like sepsis, where variability is high and protocols are less standardized, we set α to 0.5 to balance optimal and nonoptimal actions in ${\tilde{D}}_{\mathrm{so}}$. This reflects the prevalence of nonoptimal actions due to complexity and uncertainty. For conditions with more standardized treatments, a higher α would emphasize optimal actions, helping the agent replicate best practices from clinical data. Tailoring α to the task improves the agent’s practical effectiveness, and we plan to explore the impact of different α values in future work.

### Phase 2: IRL using ranked policies

In phase 2, we aim to estimate a reward function S×A→R that reflects the clinical decision quality. To achieve this, we introduce an IRL technique that effectively learns from imperfect observational data by analyzing clinician policies categorized by optimality in phase 1. Since multiple reward functions could explain the observed clinical behavior, our approach employs a machine learning model to approximate one such reward function that best reflects the variations in decision quality across these clinician policies.

First, we generate sets of trajectories that reflect varying level levels of decision quality, categorized as optimal, suboptimal, and nonoptimal. To generate these trajectories, we apply the 3-ranked policies utilize the ranked policies from phase 1—optimal ($\Pi_{\mathrm{op}}$), suboptimal ($\Pi_{\mathrm{so}}$) and nonoptimal ($\Pi_{\mathrm{no}}$)—to an independent set of patient states drawn from expert observational data, $D_{T}$, which is distinct from the dataset $D_{e}$ used in phase 1. This separation ensures the learning process mirrors genuine expert behavior, reducing potential bias and overfitting, thereby enhancing the robustness and generalizability of the learned reward function.

Each trajectory τ, is a sequence of state-action pairs:


$$\tau =\left\{\left(s_{0},a_{0}\right),\;\left(s_{1},a_{1}\right),\;\ldots ,\;\left(s_{T},a_{T}\right)\right\}\in D_{T},$$

where $s_{i}$ represents the patient’s state at time step i, and $a_{i}$ is the clinician’s action at that state. For each state $s_{i}\in D_{T}$, we generate trajectories using each of the ranked policies $\Pi_{\mathrm{op}}$, $\Pi_{\mathrm{so}}$, and $\Pi_{\mathrm{no}}$ as follows:


$$\begin{array}{l} \tau_{\Pi_{\mathrm{op}}}=\left\{\left(s_{0},\;\Pi_{\mathrm{op}}\left(s_{0}\right)\right),\;\left(s_{1},\;\Pi_{\mathrm{op}}\left(s_{1}\right)\right),\;\ldots ,\;\left(s_{T},\;\Pi_{\mathrm{op}}\left(s_{T}\right)\right)\right\},\\ \begin{array}{l} \tau_{\Pi_{\mathrm{so}}}=\left\{\left(s_{0},\;\Pi_{\mathrm{so}}\left(s_{0}\right)\right),\;\left(s_{1},\;\Pi_{\mathrm{so}}\left(s_{1}\right)\right),\;\ldots ,\;\left(s_{T},\;\Pi_{\mathrm{so}}\left(s_{T}\right)\right)\right\},\\ \tau_{\Pi_{\mathrm{no}}}=\left\{\left(s_{0},\;\Pi_{\mathrm{no}}\left(s_{0}\right)\right),\;\left(s_{1},\;\Pi_{\mathrm{no}}\left(s_{1}\right)\right),\;\ldots ,\;\left(s_{T},\;\Pi_{\mathrm{no}}\left(s_{T}\right)\right)\right\}, \end{array} \end{array}$$

where the optimal ($\tau_{\Pi_{\mathrm{no}}}\in D_{\mathrm{no}})$, suboptimal ($\tau_{\Pi_{\mathrm{so}}}\in D_{\mathrm{so}}$), and nonoptimal ($\tau_{\Pi_{\mathrm{op}}}\in D_{\mathrm{op}}$) trajectories are generated by applying the corresponding policies on the states $s_{i}\in D_{T}$. We rank these trajectories based on presumed quality of decisions, ordering them as $\tau_{\Pi_{\mathrm{no}}}<\tau_{\Pi_{\mathrm{so}}}<\tau_{\Pi_{\mathrm{op}}}.$

This ranking system supports the reward function’s ability to discern and value the nuances of decision-making, effectively reflecting the principles of optimal clinical decisions.

#### Reward function recovery

We use ranked trajectories to approximate a reward function $R_{\theta }$ parameterized by θ that guides the agent learning process. The reward function assigns cumulative rewards to each trajectory, encouraging the model to prefer trajectories with higher decision quality. For each policy $\Pi_{x}$ (where x can be optimal, suboptimal, or nonoptimal), the expected return $J\left(\Pi_{x}|R_{\theta }\right)$ is calculated as follows:


$$J\left(\Pi_{x}|R_{\theta }\right)=E_{\tau_{x}}=\left[\sum_{t=0}^{T}\gamma^{t}R_{\theta }(s_{t},a_{t})\right],$$

where γ is the discount factor and $R_{\theta }\left(s_{t},a_{t}\right)$ represents the reward assigned to the state-action pair at time t. To ensure that higher ranked trajectories receive higher rewards, we enforce the following condition:


$$J\left(\Pi_{\mathrm{no}}|R_{\theta }\right)<J\left(\Pi_{\mathrm{so}}|R_{\theta }\right)<J\left(\Pi_{\mathrm{op}}|R_{\theta }\right).$$

To learn the reward function parameters θ, we define a loss function that combines contrastive and cross-entropy losses, encouraging the model to assign higher cumulative rewards to higher ranked trajectories. For any pair of ranked trajectories ($\tau_{i}<\tau_{j}$), contrastive and cross-entropy losses are computed as follows:

*Contrastive loss* ($L_{\mathrm{CT}}$):


$$L_{\mathrm{CT}}=-\log\left(\sigma (C_{\tau_{j}}-C_{\tau_{i}})\right),$$

where σ is the sigmoid function, and $C_{\tau }=\sum_{t=0}^{T}R_{\theta }\left(s_{t},a_{t}\right)$ is the cumulative reward for trajectory τ. This loss encourages the reward function to effectively discriminate between the cumulative rewards of differently ranked trajectories, allowing higher ranked trajectories to have greater cumulative rewards than that of lower ranked trajectories.

*Cross-entropy loss* ($L_{\mathrm{CE}}$):


$$L_{\mathrm{CE}}=-\left(I\left[C_{\tau_{j}}>C_{\tau_{i}}\right]\log\hat{p}+\left(1-I\left[C_{\tau_{j}}>C_{\tau_{i}}\right]\right)\log\left(1-\hat{p}\right)\right),$$

where $\hat{p}=\sigma \left(C_{\tau_{j}}-C_{\tau_{i}}\right)$ and the indicator function $I\left[C_{\tau_{j}}>C_{\tau_{i}}\right]$ is defined as follows:


ICτj>Cτi=1 if Cτj>Cτi,0 otherwise. 

This loss penalizes incorrect ordering of cumulative rewards.

The overall optimization function for trajectory-based reward learning integrates these losses:


$$L\left(\theta \right)=L_{\mathrm{CT}}+{\beta L}_{\mathrm{CE}},$$

where $\beta \in \left[0,1\right]$ balances contrastive and cross-entropy components.

Unlike standard IRL, proposed technique enables a more precise estimation of $R_{\theta }$, enhancing our understanding of reward dynamics across ranked policies and their treatment decisions.

#### Optimal policy learning

We utilize deep Q-learning^9^^,^^60^ to learn an optimal policy using $R_{\theta }$ and the following loss function:


$$L\left(\Theta \right)=\sum \left[{\left(y-Q\left(s,a;\Theta \right)\right)}^{2}\right],$$

where Θ represents neural network parameters, and $y=R_{\theta }\left(s,a\right)+\gamma Q(s^{\prime },a^{\prime };\Theta^{-})$, where $\Theta^{-}$ are the target network parameters, updated periodically to stabilize training, and $s^{\prime }$ and $a^{\prime }$ are the next state and action.

Unlike traditional RL which relies on sparse metrics like mortality or error-prone intermittent rewards, by utilizing the approximated reward function $R_{\theta }$, we align the policy learning with the inferred reward structure, which is more closely related to practical clinical objectives.

## Nonoptimal sample selection schemas

In collaboration with clinical experts, we introduce 3 nonoptimal sample selection schemas to systematically identify suboptimal samples in sepsis and diabetes.

### Basic sepsis assessment schema (*Sepsis*^1^)

We utilize 3 health indicators for sepsis patients—Sequential Organ Failure Assessment (SOFA) score, lactate levels, and mean blood pressure (MBP)^1^^,^^61^^,^^62^—to classify observational data into nonoptimal (class no) and other (class op):


st+1|st,at=no, if st+1SOFA>0 ∧ st+1SOFA>stSOFA,     ∧st+1lactate≥4 ∧ st+1lactate>stlactate,      ∧st+1mbp<70 ∨ st+1mbp>80, op, otherwise, 

where $s_{t+1}^{\mathrm{SOFA}},\;s_{t+1}^{\mathrm{lactate}}$, and $s_{t+1}^{\mathrm{mbp}}$ are the immediate postaction SOFA score, lactate level, and MBP. Class no samples form the ${\tilde{D}}_{\mathrm{op}}$ dataset.

### Comparative sepsis assessment schema (*Sepsis*^2^)

*Sepsis* ^1^ and similar methods (popularly used as intermittent rewards in the literature) often inadequately assess optimal clinical decisions during transitions, penalizing actions that immediately worsen patient conditions despite being the best among available treatments. This obscures the distinction between truly optimal and suboptimal actions. *Sepsis*^2^ improves assessment by conducting comparative analysis within treatment contexts, penalizing actions only when demonstrably superior treatments exist. A composite score is defined for each transition to facilitate this analysis:


$$C\left(s_{t+1}|s_{t},a_{t}\right)=I_{\mathrm{SOFA}}\left(s_{t}\right)+I_{\mathrm{lactate}}\left(s_{t}\right)+I_{\mathrm{mbp}}\left(s_{t}\right),$$

where each indicator function is as follows:


ISOFA(st)={1, if st+1SOFA>stSOFA0, otherwise, 

Ilactate(st)={1, if (st+1lactate>stlactate) ∧ (st+1lactate>2)0, otherwise, 

Imbp(st)={1, if (st+1mbp<stmbp) ∧ (st+1mbp<65)0, otherwise. 

We use hierarchical clustering to categorize patient states $s_{t}$ based on physiological similarities and evaluate the optimality of treatments using the distribution of composite scores within each cluster. Treatment actions are classified into no and op classes as follows:


st+1|st,at=no, if Cst+1|st,at>Cist¯op, otherwise. ,

where $\bar{C_{i}\left(s_{t}\right)}=\sum_{s_{t}\in C_{i}}C\left(s_{t+1}|s_{t},a_{t}\right)/|C_{i}|$ is the average composite score for cluster $C_{i}$, calculated using all transitions originating from states within cluster ($s_{t}\in C_{i}$). This method directly compares nonoptimal classifications to the cluster’s average performance, refining the selection schema to accurately reflect decision outcomes in context.

We also utilized *Sepsis*^1^ to maintain consistency with baseline methods employing intermittent rewards and to demonstrate SI’s robustness across schemas.

### Glucose control assessment schema (*Diabetes*^1^)

Given the primary goal of type 2 diabetes mellitus (T2DM) management is glucose control, we devise an intuitive nonoptimal sample selection schema based on changes in hemoglobin A1c (HbA1c) and fasting blood glucose (FBG).^63^^,^^64^ Employing a cautious approach similar to sepsis, we classify treatments into no and op classes:


st+1|st,at=no, if st+1HbA1c>stHbA1c ∧ st+1fbg>stfbgop, otherwise. ,

where $s_{t+1}^{\mathrm{HbA}1c}$ and $s_{t+1}^{\mathrm{fbg}}$ are HbA1c and FBG levels at the immediate next state $s_{t+1}$ after action $a_{t}$ on state $s_{t}$. An alternative approach similar to *Sepsis*^2^ could help identify superior nonoptimal treatments. However, this ensures fair comparisons with baselines using similar mechanisms to develop intermittent rewards with HbA1c levels and FBG in the literature. Thus, any improvements can be attributed to the effectiveness of the SI learning process.

## Illustrative examples

We demonstrate the efficacy of the proposed SI using sepsis and T2DM use cases. Sepsis, a severe infection causing life-threatening acute organ dysfunction, is the third leading cause of global mortality and the primary cause of hospital deaths.^65^^,^^66^ Treatment typically involves controlling the infection source, administering antibiotics, intravenous fluids (IV), and vasopressors. However, current IV and vasopressor practices can be harmful for some patients.^13^^,^^67^ Conversely, T2DM is characterized by elevated blood glucose levels that result in significant complications and was responsible for 3.7 million deaths in 2012.^68^ Managing diabetes entails lifelong control using oral anti-diabetic drugs (OAD) and insulin. The dichotomy of sepsis and diabetes as acute and chronic diseases showcases SI’s robustness in providing both short- and long-term treatment recommendations.

### Datasets

#### Sepsis

We extracted 12 305 patients who met the Sepsis-3 criteria^69^ from the MIMIC-IV database (https://physionet.org/content/mimiciv/0.4/). The dataset includes records from 24 h prior to 48 h postdiagnosis, capturing early interventions (see Table 1). We used stratified sampling to ensure consistent mortality rates, with each patient’s entire trajectory assigned to either the training (75%) or testing (25%) set. The training set was further randomly split into $D_{e}$ (30%) and $D_{T}$ (70%).

**Table 1.** Summary statistics for sepsis and diabetes cohorts.

Cohort	Subcohort	Female (%)	Median age	ICU hours/median no. of visits	No. of patients
Sepsis	Survived	48.3	67.0	92.3	9642
	Deceased	46.4	71.0	158.5	2663
Diabetes	High HbA1c	52.5	58.0	7	1211
	Normal HbA1c	59.7	60.0	7	390

#### Diabetes

We extracted 1601 patients diagnosed with T2DM between 2011 and 2018 from Khoo Teck Puat Hospital, a tertiary care hospital in Singapore (see Table 1). We selected all patients with a minimum of 2 visits to the hospital for the study. We applied stratified sampling to ensure similar distributions of glycemic control (HbA1c levels above/below 7%) across patientwise divided training (75%) and testing (25%) sets.

No censored data (ie, cases where the full outcome is not observed within the study period) was encountered in either the sepsis or diabetes cohorts. The sepsis cohort had complete mortality information, and the diabetes cohort included patients with at least 2 hospital visits, with HbA1c levels recorded for each patient.

### Clinical problem

For the sepsis cohort, interventions (ie, IV fluids and vasopressors) are administered at 4-h intervals during the critical 24-48 h postdiagnosis. The key outcomes are mortality reduction and improved organ function. In the diabetes cohort, treatment adjustments involving OADs and insulin are made at each clinical visit, with decision timepoints based on patient visits. The primary outcomes focus on maintaining HbA1c below 7% and preventing severe hyperglycemia.

### Feature preprocessing

#### Sepsis

We identified 48 features encompassing demographics, lab values, vital signs, and intake/output events, which were aggregated into 4-hourly intervals using means or sums (see Table 2). Missing values were imputed using the last known values. Binary features were encoded as −0.5 and 0.5, while continuous values were scaled between 0 and 1 using log-normalization.

**Table 2.** Model features for sepsis cohort.

Category	Feature name
Demographics (9)	Age, Elix., shock index, SOFA, GCS, weight, SIRS, gender, readmission
Vital signs (10)	HR, SBP, MBP, DBP, Resp, Temp., PaCO_2_, PaO_2_, PaO_2_/FiO_2_ ratio, SpO_2_
Lab values (24)	Albumin, pH, calcium, glucose, Hb, magnesium, WBC, creatinine, bicarbonate, sodium, CO_2_, lactate, chloride, platelets, potassium, PTT, PT, AST, ALT, BUN, INR, ionized calcium, total bilirubin, base excess
Output events (2)	Fluid output (4 hourly), total output
Ventilation and others (3)	Mechanical ventilation, FiO_2_, timestep

Abbreviation: DBP, diastolic blood pressure; Elix., Elixhauser score; GCS, Glasgow coma scale; Hb, hemoglobin; HR, heart rate; ICU, intensive care unit; INR, international normalized ratio; MBP, mean blood pressure; PT, prothrombin time; PTT, partial thromboplastin time; Resp., respiratory rate; SBP, systolic blood pressure; SIRS, systemic inflammatory response syndrome; Temp., temperature; WBC, white blood cell.

#### Diabetes

We selected 21 relevant features including demographics, laboratory values, physiological measures, and chronic conditions (see Table 3). Data were aggregated by median for each inpatient hospital visit. Missing values were filled using the last available data from previous inpatient visits.

**Table 3.** Model features for diabetes cohort.

Category	Feature name
Demographics (4)	Age, gender, ethnicity, education level
Physiological measures (5)	SBP, DBP, height, weight, waist
Lab values (7)	HbA1C, FBG, TC, HDL, LDL, triglycerides, creatinine
Chronic condition and others (5)	Retinopathy, hypertension, hyperlipidemia, smoking history, use of lipid-lowering agent

Abbreviations: DBP, diastolic blood pressure; FBG, fasting blood glucose; HbA1c, hemoglobin A1c; HDL, high-density lipoprotein; LDL, low-density lipoprotein; SBP, systolic blood pressure; TC, total cholesterol.

### Action discretization

#### Sepsis

We discretized IV and vasopressor dosages into 5 levels, resulting a 5 × 5 action space, with level 0 indicating no drugs and levels 1-4 based on quartile dosages.

#### Diabetes

We considered 3 insulin types (basal, prandial, and premix), and 5 OAD types (metformin, sulfonylureas/glitinides, DPP4, α-glucosidase inhibitors, and thiazolidinediones) and defined a 4 × 5 action space to represent combinations of drugs administered.

Dataset extraction, feature selection, preprocessing, and action discretization followed common practices.^1^^,^^41^^,^^70^

### Baselines

We compared the proposed SI against following state-of-the-art deep RL and IL baselines.

- **D3QN** ^1^ **:** State-of-the-art deep RL solution for sepsis, utilizing the same features and Dueling Double Deep Q Learning (D3QN) architecture as SI. We term the baseline as D3QN, following its architecture. D3QN is a crucial benchmark to highlight improvements from the proposed 2-phase learning process. We used the original implementation (https://github.com/darkefyre/sepsisrl/) with tuned hyperparameters to fit the cohorts. D3QN employs mortality-based and clinically crafted intermittent rewards, like the Sepsis^1^ criteria.
- **NFQ:** Deep RL agent using neural fitted Q iteration, adapted for ICU treatments.^71^^,^^72^ We implemented NFQ with D3QN’s reward function for direct comparisons against established solutions.
- **POfD** ^54^ **:** Based on GAIL, POfD directly imitates imperfect expert observational data and uses standard RL to explore unseen scenarios, thus highlighting the need for solutions that address demonstrator imperfections. Originally designed for gaming, we adjusted POfD’s state and action definitions for treatment recommendation.
- **IC-GAIL** ^55^ **:** State-of-the-art GAIL based game playing agent that achieves demonstrator-level optimality using occupancy measure matching, relying on an oracle for optimality assessment. Due to the impracticality of oracles in healthcare, we used the proposed Sepsis^2^ schema to help distinguish and deviate from nonoptimal samples. However, IC-GAIL exploration is limited to scenarios within expert observational data.
- **ACIL** ^24^ **:** Enhances GAIL with a cooperative discriminator and a model-based RL framework. ACIL strictly adheres to survival trajectories, inaccurately assuming all treatments within them are optimal and vice versa. Additionally, it relies heavily on a trained environment model, which can be catastrophic given the limited data quality in healthcare.
- **SI-S1, SI-S2, and SI-D:** Proposed SI variants trained using Sepsis^1^, Sepsis^2^ and Diabetes^1^ schemas. (See Appendix S1 for algorithm pseudocode, model architecture, and parameters.)

## Results

We conducted extensive analyses to evaluate the performance of the SI framework against baseline models. Our evaluation strategy combined both quantitative and qualitative metrics to provide a comprehensive assessment of the learned policies’ effectiveness, safety, and potential clinical impact in managing both sepsis and T2DM. This study aims to address the following research questions.

### RQ1: can the proposed SI framework effectively learn treatment policies from imperfect observational data and outperform existing baseline models?

To answer **RQ1**, we conducted extensive off-policy evaluations (OPEs) to compare SI with baseline models. Treatment recommendation is an off-policy learning problem deriving an optimal policy using clinician trajectories. Off-policy evaluation uses existing clinical decisions to gauge the effectiveness and safety of learned policies without deploying in real world.

#### OPE evaluation metrics

We employed a combination of quantitative metrics to assess the learned policies:

1. Consistent weighted per-decision importance sampling Effective sample size (CWPDIS): This metric measures the expected returns of the learned policy by adjusting for action distribution differences between the learned and clinician policies.^73^ CWPDIS offers a consistent and low-variance estimation ideal for long-horizon issues typical in healthcare. It adjusts expected returns on a per-decision basis and provides more stable estimates than traditional importance sampling methods (see Appendix S1, Section 3.1 for the formula). A higher CWPDIS value indicates effective replication of clinician subtrajectories with high rewards.
2. Effective sample size (ESS): It quantifies the amount of useful information for evaluation by measuring the steps in overlapping subtrajectories between learned and clinician policies. A higher ESS indicates greater confidence in evaluation outcomes. The clinician ESS, which counts interventions in the test set, serves as the maximum achievable ESS.
3. Clinical outcome metrics: For sepsis, we calculated mortality rates by categorizing patient trajectories based on expected returns and computing the average mortality for each category. An optimal policy should result in lower mortality rates, indicating improved patient survival. For diabetes, we computed HbA1c-High rates, defined as the percentage of patients with HbA1c levels ≥7% at treatment end. Lower HbA1c-High rates indicate better glycemic control and treatment effectiveness.

While alternative OPE metrics such as doubly robust estimators,^74^ inverse probability weighting (IPW),^15^ and the direct method (DM)^75^ exist, they have limitations in healthcare applications. Doubly Robust Estimators leverage the “double robustness” property, ensuring consistency when either the propensity score or the outcome model is correctly specified. However, their practical utility in healthcare is limited due to the difficulty of accurately estimating either model in environments with complex, high-dimensional, or sparse data. Inverse probability weighting is prone to high variance, making it less effective for long patient trajectories and rare events common in healthcare. Direct method heavily depends on the accuracy of the estimated reward model, which may be unreliable in complex healthcare environments. By selecting CWPDIS, ESS, and clinical outcome metrics, we ensure a robust and reliable OPE, better suited to the intricacies of healthcare data, where sparse or noisy information, small subcohorts, and heterogeneity in clinician behaviors are common.

#### Results in sepsis management

Table 4 presents the OPE results for the sepsis cohort. SI-S2 achieved the highest CWPDIS of 20.40, outperforming the closest baseline, NFQ (12.80), by 59.38%. Similarly, SI-S1 recorded a CWPDIS of 19.66, which was 53.59% higher than NFQ. The ESS for SI-S2 and SI-S1 were 860 and 733, demonstrating strong alignment with clinician actions. In contrast, D3QN achieved the highest ESS of 1266 but failed to replicate optimal trajectories effectively due to a low CWPDIS of 3.47.

**Table 4.** Off-policy evaluation for sepsis.

Model	ESS	CWPDIS	Mortality (%)
**Clinician**	37 915	9.40	21.36
**POfD**	91	3.85	18.63 ± 0.45
**IC-GAIL**	251	7.44	18.25 ± 0.67
**ACIL**	545	0.83	17.29 ± 0.22
**NFQ**	80	12.80	16.04 ± 0.56
**D3QN**	1266	3.47	14.76 ± 0.53
**SI-S1**	733	**19.66**	**12.15 ± 0.06**
**SI-S2**	860	**20.40**	**11.87 **±** 0.01**

Effective policies have higher CWPDIS and ESS, with lower mortality rates. Bold values indicate the best-performing results for CWPDIS and mortality across all models.

Abbreviations: ACIL, adversarial cooperative imitation learning; CWPDIS, consistent weighted per-decision importance sampling; ESS, effective sample size; NFQ, neural fitted Q; POfD, policy optimization from demonstration.

Mortality rates for SI-S2 and SI-S1 were 11.87% and 12.15% compared to 14.76% for the best-performing baseline, D3QN, representing reductions of 19.58% and 17.68%. These results indicate that compared to baselines, the learned policies could substantially improve the patient survival rates.

#### Results in diabetes management

Table 5 presents the OPE results for the diabetes cohort. Based on ESS, SI-D has the highest concordance with the clinician after POfD and IC-GAIL. This indicates their efficacy in mimicking observational data. However, based on CWPDIS, SI-D significantly outperforms all baselines, with CWPDIS for SI-D being 52.32% higher than the closest baseline D3QN. Despite high ESS for POfD, its tendency to strictly follow all clinician actions regardless of optimality leads to low CWPDIS. Moreover, SI-D showed a 12.25% lower mean HbA1c-High rate than the closest NFQ baseline and significantly outperformed the rest.

**Table 5.** Off-policy evaluation for diabetes.

Model	ESS	CWPDIS	HbA1c-High rate (%)
**Clinician**	5452	3.16	73.57
**D3QN**	189	8.83	73.31 ± 1.66
**ACIL**	48	8.56	71.27 ± 0.61
**IC-GAIL**	341	7.53	68.67 ± 0.31
**POfD**	357	6.21	66.44 ± 0.01
**NFQ**	282	8.63	66.35 ± 2.05
**SI-D**	302	**13.45**	**58.22 ± 0.01**

Effective policies have higher CWPDIS and ESS, with low HbA1c-High rates. Bold values indicate the best-performing results.

Abbreviations: ACIL, adversarial cooperative imitation learning; CWPDIS, consistent weighted per-decision importance sampling; ESS, effective sample size; NFQ, neural fitted Q; POfD, policy optimization from demonstration.

These results suggest a positive response to **RQ1,** demonstrating that the proposed SI framework shows potential in learning treatment policies from imperfect observational data that could outperform existing baseline models in both sepsis and diabetes management.

### RQ2: how do the expected returns of the learned policies correlate with clinical outcomes such as mortality and HbA1c-High rates?

**Figure 2.**
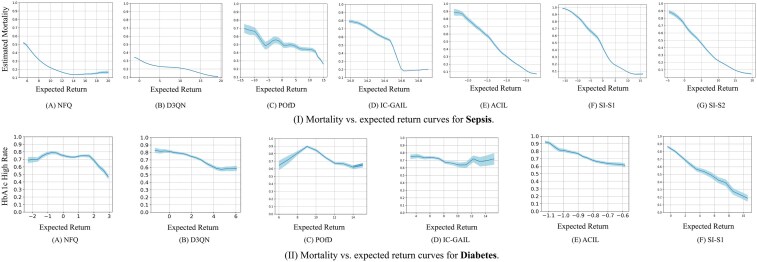
(A-G) Mortality vs expected return curves for sepsis. (H-M) HbA1c-High rates vs expected return curves for diabetes. Shaded areas indicate SDs.

To answer **RQ2**, We analyzed the correlation between mortality and expected returns for sepsis (see Figure 2I), and HbA1c-High rate and expected returns for diabetes (see Figure 2II). An effective policy should showcase a clear negative correlation, where higher expected returns correspond to lower mortality and HBA1c-High rates, and vice versa.

For sepsis, NFQ, POfD, and IC-GAIL occasionally showed positive correlations, suggesting these policies associate higher returns with trajectories having a higher risk of mortality. Conversely, D3QN, ACIL, SI-S1, and SI-S2 demonstrated consistent negative correlations, highlighting their effectiveness in guiding treatments that improve patient outcomes.

For diabetes, NFQ, POfD, IC-GAIL, and D3QN failed to consistently maintain the desired negative correlation. Conversely, ACIL and SI-D showed the desired clear negative correlation, with SI-D having the strongest negative correlation.

The results suggest that the SI framework’s learned policies exhibit a negative correlation with adverse clinical outcomes, such as mortality and HbA1c-High rates, providing support for **RQ2**.

### RQ3: do the treatment recommendations generated by the SI framework align with clinician practices across different patient severity levels, and where do they diverge?

To answer **RQ3**, we compared the treatment recommendations from the SI with clinician practices across several patient severity levels to assess the alignment and potential improvements in treatment strategies.

#### Analysis of mortality and HbA1c-High rates against dosage differences

Sepsis patients were categorized into low (<5), medium (5-15), and high (>15) severity groups based on SOFA scores, while diabetes patients were classified into low (<7), medium (7-8), and high (>8) severity levels based on HbA1c values. We analyzed mortality (and HbA1c-High rates) against dosage differences for each severity level. An effective policy should show a V-shaped correlation, with the lowest mortality (HbA1c-High rates) at zero dosage difference, increasing with higher dosage discrepancies. Due to space limitations, we illustrate SI-S1, SI-S2, and SI-D against the best-performing baseline for each group, since no baseline was consistent across all groups (see Appendix S1, Section 4 for full results).

**Figure 3.**
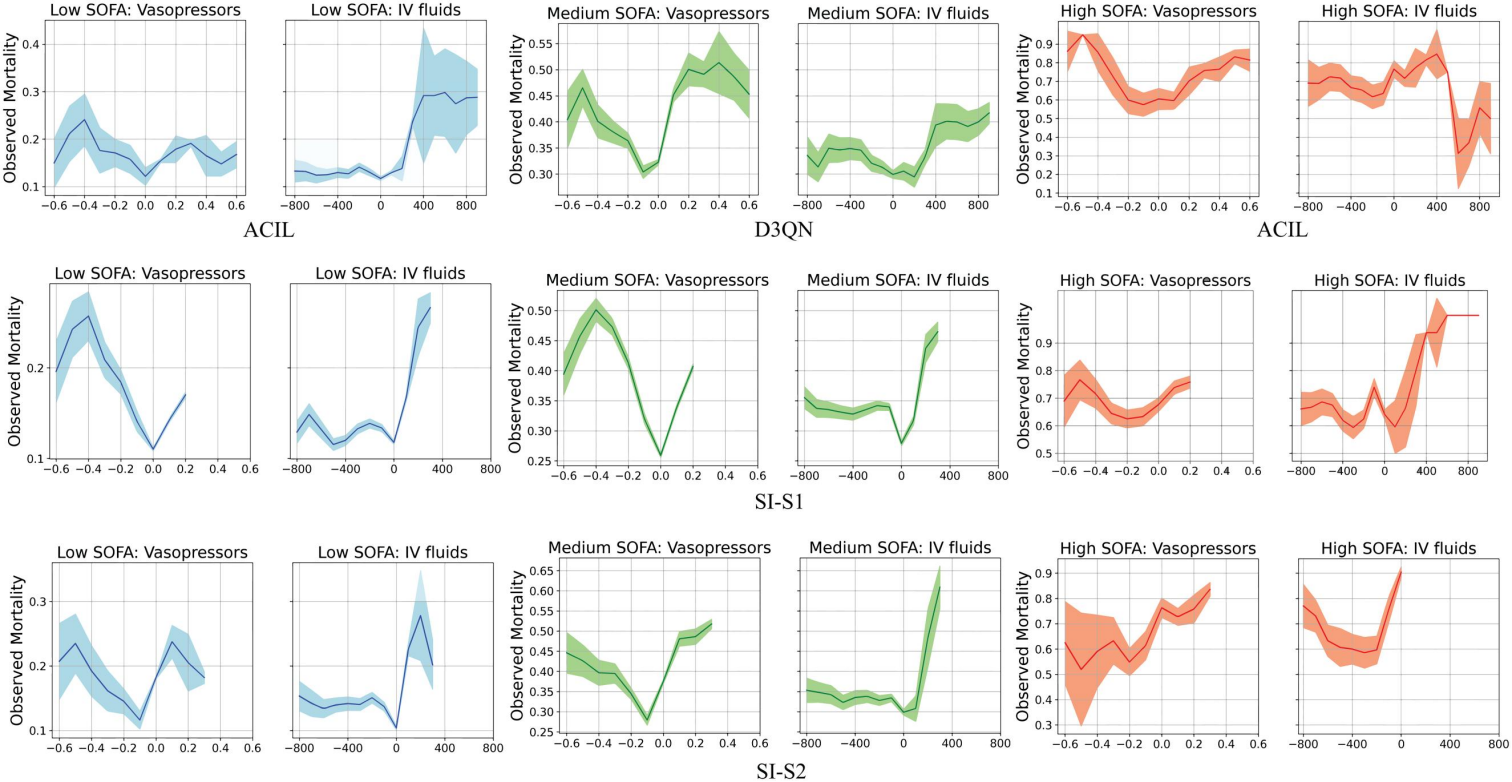
Changes in mortality (*y*-axis) vs differences between optimal policy recommended and clinician-administrated dosages (*x*-axis) for sepsis. Graphs compare proposed SI with best-performing baseline for low (blue), medium (green), and high (red) SOFA levels. Abbreviations: SI, Smart Imitator.

For sepsis, among the baselines, ACIL performed best at low and high SOFA levels, while D3QN was most effective at medium levels (see Figure 3). All baselines were less effective at high SOFA levels. In contrast, SI-S1 and SI-S2 closely followed the anticipated V-shape across severity levels, except when treating high-risk patients with specific recommendations: SI-S1 with vasopressors and SI-S2 with IV treatments—likely due to fewer high-severity samples. Despite these challenges, SI models consistently outperformed baselines.

**Figure 4.**
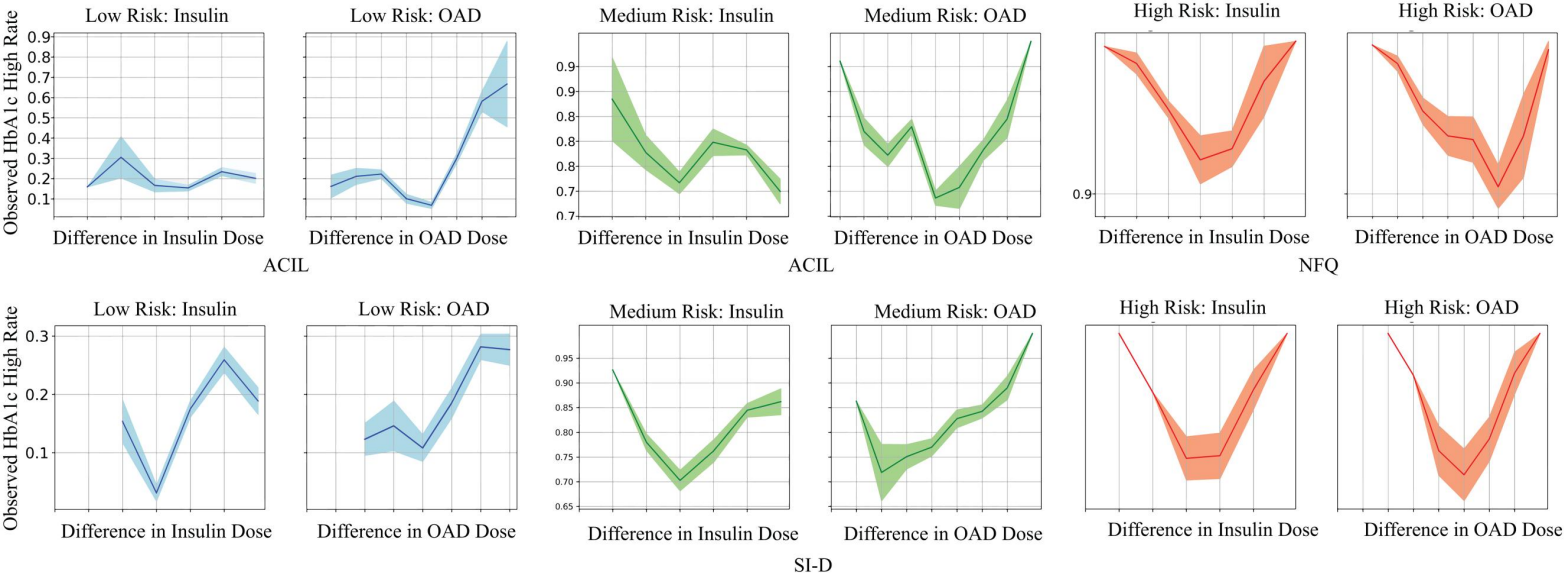
Changes in mortality (*y*-axis) vs differences between optimal policy recommended and clinician-administered dosages (*x*-axis) for diabetes. Graphs compare proposed SI with all models for low (blue), medium (green), and high (red) HbA1c values. Abbreviations: SI, Smart Imitator.

For diabetes, all baselines were suboptimal for low and medium severity levels, with only ACIL partially achieving the desired V shape (see Figure 4), and NFQ was the best baseline for high severity levels with a horizontally shifted V shape for OAD treatments. In contrast, SI-D showed the desired V shape for all severity levels, except for OAD treatments, where the V shape was slightly shifted for medium and high severity levels. SI-D significantly outperformed all baselines across all severity levels.

#### Clinician vs learned policies

**Figure 5.**
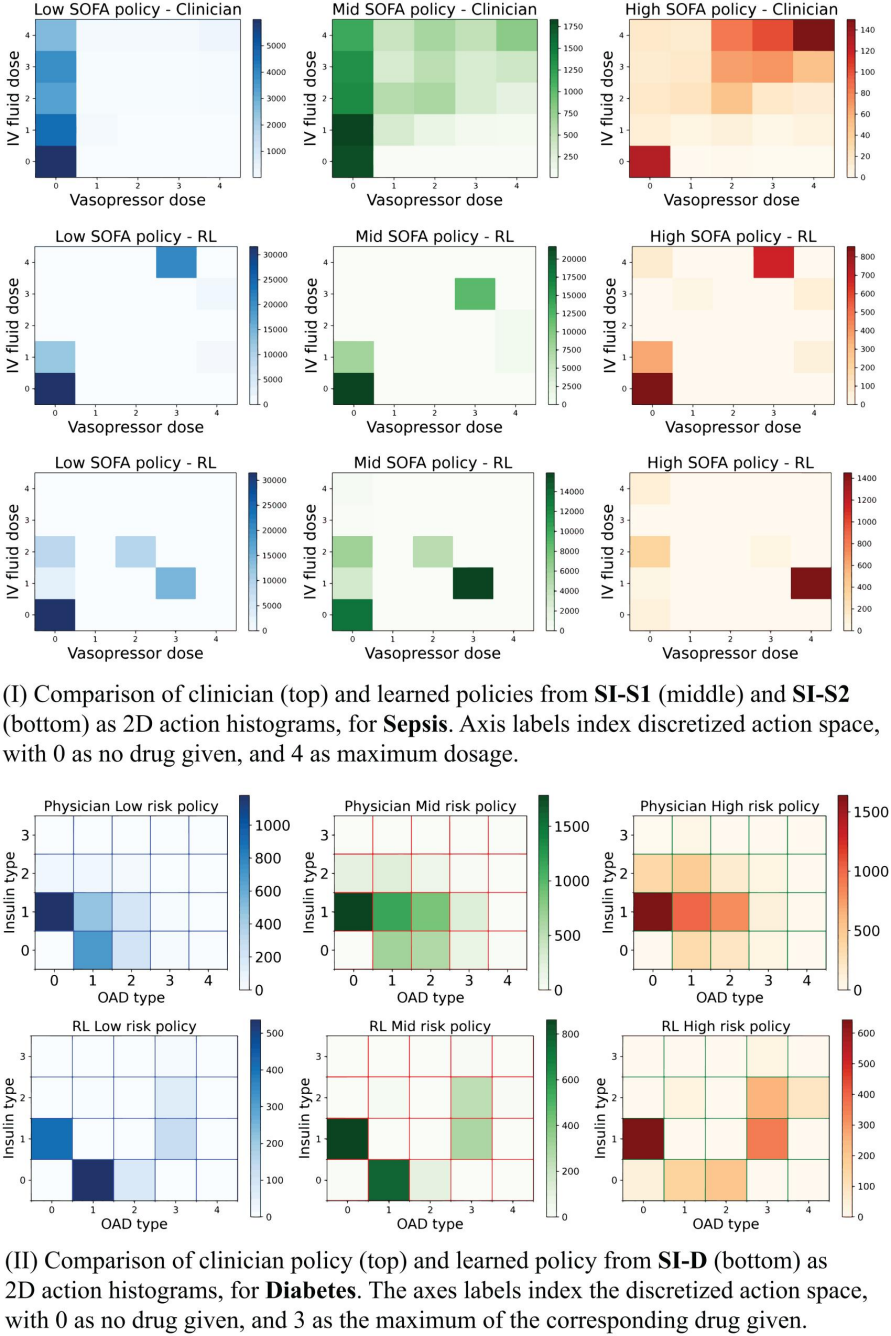
Comparison of clinician and learned policies from SI-S1, SI-S2, and SI-D as 2D action histograms, for sepsis and diabetes, respectively.

For sepsis, we compared clinician treatments against SI-S1 and SI-S2 recommendations for 25 action combinations (5 × 5 dosages) (see Figure 5I). At low and medium SOFA levels, clinicians generally recommend minimal medications, typically no medication or only IV, with increasing vasopressor use as severity increases. For high SOFA levels, a combination of IV and vasopressors is frequent. Similarly, learned policies suggest no medication or IV at lower severities, progressively recommending more vasopressors and a mix of both treatments at high severity levels. While following similar trends, SI-S1 and SI-S2 recommend higher vasopressor usage, a data-driven adjustment supported by clinical trials suggesting improved outcomes with increased vasopressor use.^76^

For diabetes, we compared clinician treatments against SI-D recommendations for 20 action combinations (4 × 5 dosages) (see Figure 5II). Contrary to sepsis, where clinicians recommended minimal drugs, clinicians consistently administered insulin and/or OADs with a slightly higher preference for insulin. Dosages typically increased with patient severity. The learned policy recommends similar treatments with a slight increase in OAD for all severity levels. This indicates the learned policy follows clinical decisions with necessary adjustments to achieve better HbA1c-High rates.

In conclusion, the treatment recommendations from the SI framework largely align with clinician practices across different patient severity levels, with observed potentially indicating areas of improvements through data-driven insights, supporting **RQ3**.

### RQ4: does the learned reward function in the SI framework offer advantages over traditional hand-engineered reward functions in guiding policy learning and improving patient outcomes?

**Figure 6.**
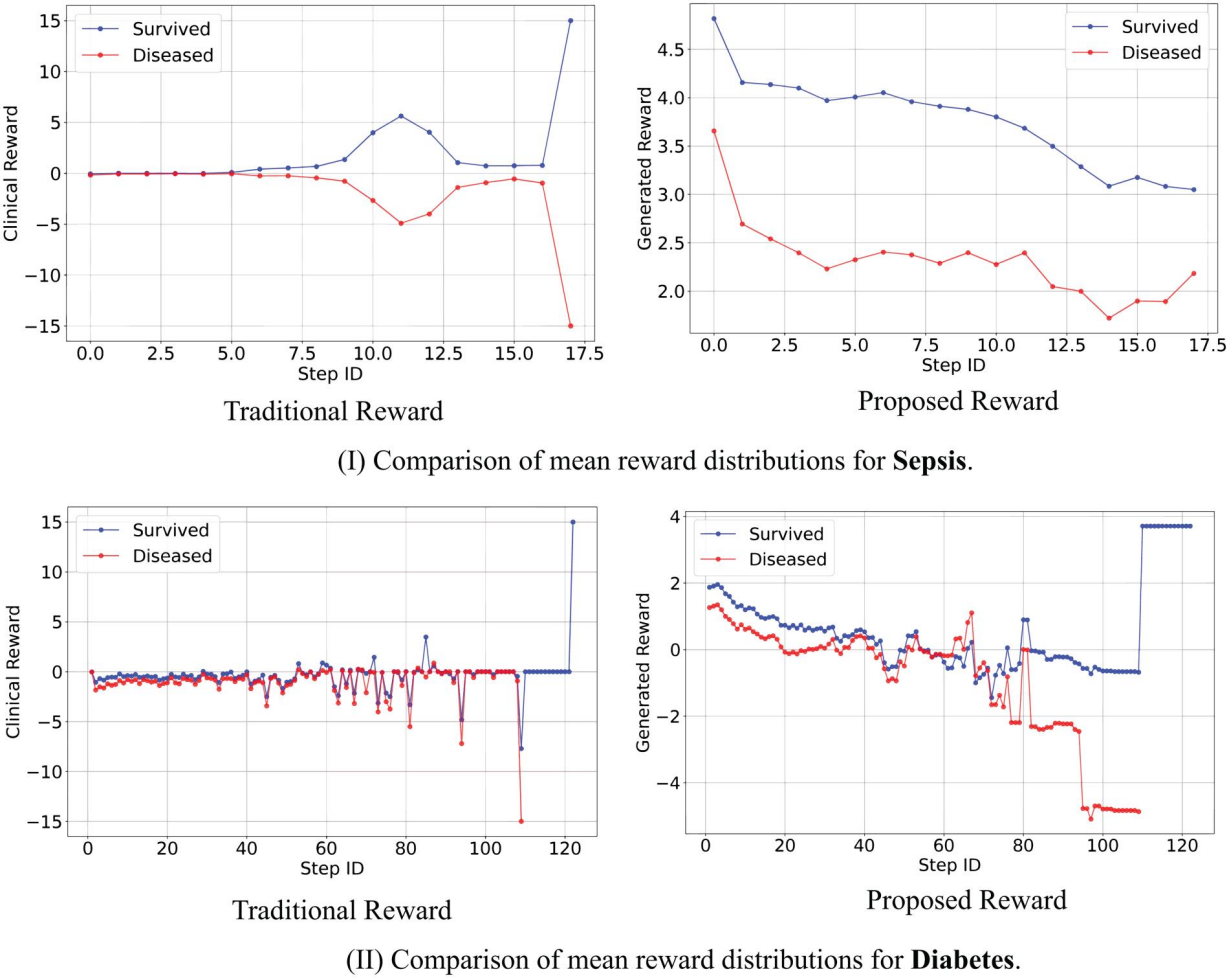
Comparison of mean reward distributions of traditional and proposed reward functions for sepsis and diabetes treatments.

To answer **RQ4**, we evaluated learned reward functions against traditional hand-engineered functions,^1^^,^^77^ analyzing average rewards from admission to discharge for survived and deceased patients (see Figure 6). Unlike traditional functions that heavily rely on terminal states for significant rewards/penalties, learned reward functions provide granular and consistent feedback from intermediate states, mitigating reward sparsity and complex credit assignment issues. Notably, although not explicitly trained on patient mortality, the learned function assigns higher rewards to survival trajectories, indicating its ability to associate positive actions with better outcomes.

## Discussion

### Interpretation of results

#### RQ1: effectiveness of SI framework in learning optimal policies

The results demonstrate the SI framework’s superior ability to learn effective treatment policies from imperfect observational data. High CWPDIS values and reduced adverse outcomes affirm the framework’s capacity to replicate optimal clinician subtrajectories while avoiding suboptimal ones. In comparison, RL baselines such as D3QN and NFQ faced significant challenges in learning optimal policies. These limitations stem from exploration complexities unique to treatment recommendations, such as long-horizon reward sparsity and the presence of suboptimal actions within clinician trajectories.

For example, the low CWPDIS observed in D3QN, despite high ESS, highlights its inability to distinguish between optimal and suboptimal strategies. Similarly, ACIL’s design, which strictly follows all clinician actions linked to survival outcomes, fails to address suboptimal actions embedded in successful trajectories. This discrepancy is consistent with previous findings that suboptimal actions are often prevalent in expert observational data, even in cases with favorable outcomes.^14^^,^^25–27^ In contrast, the SI framework manages these complexities effectively, leveraging ACIL and IRL to derive superior policies.^5^^,^^78^

Similar patterns were observed in diabetes management, where traditional RL baselines such as D3QN and NFQ struggled with exploration complexities and high reward sparsity, resulting in suboptimal policy learning. The SI framework, by addressing these challenges, consistently outperformed baseline models across both acute and chronic disease contexts.

#### RQ2: correlation between expected returns and clinical outcomes

The SI framework’s ability to achieve consistent negative correlations between expected returns and adverse outcomes, such as mortality and HbA1c-High rates, highlights its alignment with improved clinical outcomes. In the sepsis cohort, the negative correlation observed for SI-S1 and SI-S2 underscores their ability to guide effective treatments that reduce mortality. On the other hand, traditional baselines such as NFQ and IC-GAIL exhibited occasional positive correlations, linking higher returns to worse outcomes, which is indicative of suboptimal policy learning. Similarly, in the diabetes cohort, the SI-D model demonstrated the strongest negative correlation between expected returns and HbA1c-High rates, outperforming all baselines. These results suggest that the SI framework successfully captures the nuances of treatment efficacy, making it a reliable tool for optimizing clinical interventions.

#### RQ3: alignment with clinician practices across severity levels

The SI framework aligns closely with clinician practices while introducing data-driven optimizations to improve patient outcomes. Across severity levels, SI models consistently outperformed baselines by aligning with clinician actions that reduced mortality and avoided less effective strategies. In the sepsis cohort, SI-S1 and SI-S2 adhered to the anticipated V-shaped correlation between mortality rates and dosage differences, even in high-severity cases with sparse data. By recommending vasopressors and IV treatments in high-risk scenarios, SI models introduced adjustments supported by clinical evidence, which further improved patient outcomes. Similarly, in the diabetes cohort, SI-D maintained a V-shaped correlation for HbA1c-High rates and dosage differences across all severity levels. While slight deviations were observed in OAD treatments at medium and high severities, SI-D consistently optimized dosages to achieve better glycemic control.

#### RQ4: advantages of learned reward functions

The learned reward functions in the SI framework offer significant advantages over traditional hand-engineered reward functions. By providing granular feedback throughout patient trajectories, the learned rewards mitigate common challenges in healthcare RL, such as reward sparsity and complex credit assignment. Both qualitative and quantitative assessments support the notion that these functions enhance survival rates and promote more effective treatment policies. Unlike conventional methods that heavily depend on terminal states for reward signals, the SI framework assigns higher rewards to survival trajectories without explicit reliance on mortality labels. This ability to guide policy learning effectively positions the SI framework as a robust decision-support tool for healthcare.

### Broader implications

The SI framework has the potential to transform healthcare decision-making by addressing key challenges posed by imperfect observational data, including confounding factors inherent in high-dimensional datasets. Unlike traditional statistical adjustment approaches, which rely heavily on covariate balance or accurate propensity score estimation, the SI framework mitigates confounding through its design. By leveraging clinically guided schemas, ACIL, and IRL, SI minimizes biases introduced by unobserved factors and sparse data. These mechanisms allow it to better align observed actions with outcomes, even when confounders are not fully captured at the data collection stage. Its ability to replicate clinician actions while introducing data-driven optimizations highlights its utility as a decision-support tool for personalized medicine. Where traditional methods often struggle with high-dimensionality or assumptions of optimality, the SI framework’s 2-phase learning process ranks clinician-derived policies by effectiveness and approximates the true reward function. This structured approach enables it to learn meaningful patterns and develop superior treatment strategies, even from suboptimal observational data.

Evaluations across acute and chronic diseases demonstrate the framework’s capacity to outperform existing methods, significantly improving patient survival and glycemic control. Metrics like CWPDIS and ESS further validate SI’s robustness in handling variability and confounding, providing actionable insights that align with clinical outcomes. These findings mark an important step toward establishing Artificial Intelligence (AI) powered healthcare as collaborative systems, effectively supporting clinician practices in complex, variable treatment environments.

### Limitations and future directions

While the SI framework demonstrates strong potential, sparse data in high-severity cases, particularly in sepsis, restricted its performance in critical scenarios. Addressing this limitation will require larger datasets with better representation of high-risk patient populations and closer collaboration with clinicians to refine the model’s ability to handle suboptimal trajectories and severe conditions.

Future work should focus on validating the SI framework through prospective clinical trials, such as SMARTs, to evaluate its real-time adaptability and effectiveness in clinical settings. Expanding its application to a broader range of diseases will also enhance its generalizability across healthcare contexts. Ultimately, integrating the framework into clinical workflows will solidify its role as a collaborative tool, supporting personalized, evidence-based treatment strategies and advancing the goals of precision medicine.

## Author contributions

Dilruk Perera made substantial contributions to the conceptualization of the study, formal analysis, investigation, methodology development, and project administration; was responsible for validating the data, preparing visualizations, and drafting the original manuscript. Dilruk Perera and Mengling Feng provided final approval of the version to be published and agreed to be accountable for all aspects of the work. Siqi Liu contributed significantly to data curation, methodology development, and the preparation of visualizations for the study; and provided final approval of the manuscript and agreed to be accountable for all parts of the work. Kay Choong See played a key role in the conceptualization of the study and data curation; acted as a clinical collaborator, assisting with data validation; and provided final approval of the manuscript and agreed to be responsible for the integrity of the entire work. Mengling Feng contributed to the conceptualization of the study, secured funding, and was responsible for project administration and the provision of resources; supervised the entire project.

## Supplementary material

Supplementary material is available at *Journal of the American Medical Informatics Association* online.

## Funding

This research/project is supported by the National Research Foundation, Singapore under its AI Singapore Programme (AISG Award No: AISG-GC-2019-001-2B) and Grant Number AISG2-TC-2022-004. This work was also supported by the RIE2025 Industry Alignment Fund, Cisco-NUS Accelerated Digital Economy Corporate Laboratory, grant number I2101E0002. Additional support was provided by the Talent Development Award 2023 from Saw Swee Hock School of Public Health, grant number 24-0180-A0001-0.

## Conflicts of interest

The authors have no competing interests to declare.

## Data availability

This study uses the MIMIC-IV database, which is publicly available at https://physionet.org/content/mimiciv/0.4/. Access to the dataset requires completion of the necessary certification and approval from the data custodians. Upon acceptance of the manuscript, we will provide all relevant code for data extraction, preprocessing, model tuning, and analysis through a publicly accessible repository to ensure reproducibility and transparency.

## Supplementary Material

ocae320_Supplementary_Data
