# Correction to: Smart Imitator: Learning from Imperfect Clinical Decisions

**DOI:** 10.1093/jamia/ocaf098

**Published:** 2025-06-15

This is a correction to: Dilruk Perera, Siqi Liu, Kay Choong See, Mengling Feng, Smart Imitator: Learning from Imperfect Clinical Decisions, *Journal of the American Medical Informatics Association*, 2025, ocae320, https://doi.org/10.1093/jamia/ocae320

The first sentence of the funding declaration has been updated from “This work was supported by the National Research Foundation, Singapore, under its AI Singapore Programme, grant numbers AISG-GC-2019-001-2B and AISG2-TC-2022-004” to “This research/project is supported by the National Research Foundation, Singapore under its AI Singapore Programme (AISG Award No: AISG-GC-2019-001-2B) and Grant Number AISG2-TC-2022-004.”

