# Supplementary material for: Smart Imitator: Learning from Imperfect Clinical Decisions
Source: J Am Med Inform Assoc. 2025 Jan 10;33(1):49–66. doi: 10.1093/jamia/ocae320 (PMC12758472; doi:10.1093/jamia/ocae320)
Supplement: ocae320_Supplementary_Data [file ocae320_supplementary_data.docx]

Smart Imitator: Appendices

1. **PRELIMINARIES**
   1. **Behavior Cloning (BC)**

BC replicates expert’s decision-making process by learning a policy $\pi_{\theta}\left( a | s \right)$ that maps states $s$ to actions $a$ based on expert observational data $\mathcal{D}_{T}= \left\{ \left( s_{1}, a_{1} \right), \left( s_{2}, a_{2} \right), . . . , \left( s_{N}, a_{N} \right) \right\}$, where each tuple $\left( s_{i}, a_{i} \right)$ is a state-action pair from expert policy $\pi_{E}(a|s)$ [1]. BC minimizes the discrepancy between the actions predicted by the learned policy ($\pi_{\theta}$) and those performed by the expert:

$$\min_{\pi_{\theta}} \sum_{\left( s_{i},a_{i} \right)\in\mathcal{D}_{T}} L\left( \pi_{\theta}\left( a|s_{i} \right),a_{i} \right)$$

where the loss function $L$ measures this divergence. BC is straightforward and effective when quality expert data is available, allowing for rapid model deployment without complex reward structures. However, it learns sub-optimal policies from imperfect observational data, suffers from compounding errors and fails to generalize to unseen states. BC is a solid starting framework that needs to be refined to improve adaptability and effectiveness.

- 1. **Inverse Reinforcement Learning (IRL)**

IRL is designed to infer the underlying reward function from expert decisions, capturing their rationale to develop robust policies that could generalize beyond given observational data $\mathcal{D}_{T}$. The IRL objective is formulated as follows:

$$\max_{R} \mathbb{E}_{\left( s_{i},a_{i} \right)\in\mathcal{D}_{T}}\left[ R\left( s,a \right) \right]$$

where $R(s, a)$ is a step wise reward function.

Subsequently, a policy $\pi_{\theta}$ is optimized using standard RL techniques to maximize the expected sum of rewards ${max}_{\pi_{\theta}}\mathbb{E}_{\pi_{\theta}}\left[ \sum_{t} R\left( s_{t},a_{t} \right) \right]$. However, using conventional IRL methods to derive effective reward functions from imperfect observational data can result in superficial outcomes. These often lead to inaccurate action interpretations and imprecise reward assessments [2–4]. However, learning an effective reward function using standard IRL techniques is challenging since traditional techniques, when applied to imperfect observational data, often result in superficial reward functions [2–4]. This leads to inaccurate interpretations of actions and imprecise assessment of relative rewards. Therefore, SI introduces a technique to accurately estimate the reward function using a novel policy ranking method and a unique loss function.

- 1. **Generative Adversarial Imitation Learning (GAIL)**

GAIL integrates IL with GANs, involving dynamic interactions between a generator $G$ and a discriminator $D$. $G$ learns a policy $\pi_{\theta}$ to replicate state-action pairs similar to expert observational data while the discriminator $D(s, a)$, functioning as a binary classifier, evaluates whether these pairs are derived from the expert policy $\pi_{E}$ or the generator. This forms a min-max adversarial game:

Integrates IL with GANs, which involves dynamic interactions between a generator G and a discriminator $D$. $G$ attempts to learn a policy $\pi_{\theta}$ that produces state-action pairs similar to expert observational data. $D(s, a)$, functions as a binary classifier that determines the origin of the given state-action pairs (expert or $G$). Interaction between these components forms a min-max adversarial game as follows:

$$\min_{\pi_{\theta}} \max_{D} \left[ \log D\left( s,a \right) \right]+\mathbb{E}_{\pi_{E}}\left[ \log\left( 1-D\left( s,a \right) \right) \right],$$

where $G$ aims to deceive $D$, while $D$ aims to accurately distinguish actions from $\pi_{E}$ and $\pi_{\theta}$.

In SI, we extend GAIL to incorporate an adversarial cooperative min-max game [5], enhancing the discriminator and generator with cooperative and discriminative elements. This extension trains $\pi_{\theta}\left( a|s \right)$ to more closely emulate optimal actions and avoid non-optimal ones.

We extend AIL to use an adversarial cooperative min-max game, with cooperative and discriminative elements within the discriminator and generator. The aim is to train a policy $\pi_{\theta}\left( a|s \right)$ that closely aligns with optimal demonstrator actions and deviates from non-optimal actions.

1. **MODEL ARCHITECTURE AND PARAMETERS**

Following best practices in RL for healthcare [6–9], we employed a Dueling DQN architecture [10] with main and target DQN networks, each having three hidden layers of 256, 128, and 64 units. This architecture was chosen for its ability to separate state-value from action-advantage, which helps mitigate overestimation bias and improves generalization in complex, noisy environments like healthcare.

To address overfitting, we applied several strategies. First, L2 regularization was used to penalize large weights, preventing the model from becoming overly complex. We also implemented dropout layers during training, which deactivated random neurons to encourage the network to learn more generalized features. Additionally, early stopping was employed to halt training when the model’s performance on the validation set plateaued, ensuring that it did not overfit to noise in the training data. Finally, we conducted cross-validation to ensure robust performance across different data splits, minimizing the risk of overfitting to any specific dataset.

We balanced adversarial and cooperative losses by setting both $\lambda_{A}$ and $\lambda_{C}$ to 0.5. The blending coefficient $\alpha$ was set at 0.5 to balance optimality and non-optimality in $\Pi_{so}$, and $\beta$ was set at 0.5 to equally weigh contrastive and cross-entropy losses in reward learning. The learning rate for phase 2 was 0.001, with terminal rewards and penalties standardized at +15 and −15. All models underwent consistent testing on the same holdout set to ensure uniformity. Codes accompanying this study will be publicly available post-acceptance.

Algorithm 1 outlines the two-phase learning process in the proposed Smart Imitator.

| **Algorithm 1** Smart Imitator: Two-Phase Policy Learning from Imperfect Observational Data. | | |
| --- | --- | --- |
|  | **Input:** | Complete Clinician Observational Data $D=\left\{ \left( s_{i},a_{i} \right) \right\}_{i=1}^{n}$,  Initial parameters for reward function ($\theta_{0}$), Policy ($\Theta_{0}$), discriminators ($\omega_{A,0}$ and $\omega_{C,0}$), and BC learning ($\psi_{no,0}$ and $\psi_{so,0}$).  Hyperparameters $\lambda_{A}$, $\lambda_{C}$, $\alpha$, $\beta$ and $\gamma$. |
|  | **Output:** | Optimal Policy $\Pi_{\Theta}$. |
|  | **Preparation Phase:** | **Data Division and Sampling.** |
|  |  | $\mathcal{D}_{train}, \mathcal{D}_{test}\leftarrow\mathrm{split}\left( \mathcal{D}, 75\%,25\% \right)$ |
|  |  | $\mathcal{D}_{e}, \mathcal{D}_{T}\leftarrow\mathrm{split}\left( \mathcal{D}_{train}, 30\%,70\% \right)$ |
|  | **Phase 1:** | **IL based Policy Ranking in Imperfect** Observational Data**.** |
|  |  | Identify and classify policies into $\Pi_{\mathrm{op}}$, $\Pi_{\mathrm{so}}$, and $\Pi_{\mathrm{no}}$ based on their optimality, using $\mathcal{D}_{T}$. |
|  | **Phase 2:** | **Inverse RL using Ranked Policies.** |
|  |  | Construct datasets $\mathcal{D}_{no}$, $\mathcal{D}_{so}$, and $\mathcal{D}_{op}$ from $\mathcal{D}_{T}$ based on the policy rankings. |
|  |  | Learn the underlying reward function $R_{\theta}$using the ranked policies. |
|  |  | Learn an optimal policy $\Pi_{\mathrm{op}}\left( \Theta\right)$ under $R_{\theta}$ using Deep Q-learning. |
|  |  |  |

1. **EXPERIMENT SETUP**
   1. **Off Policy Evaluation Metrics**

We use state-of-the-art Consistent Weighted Per-Decision IS (CWPDIS) to evaluate learnt policies.

$$CWPDIS\left( \pi_{\theta},\mathcal{D}_{T} \right)\triangleq\sum_{t=1}^{T} \frac{\sum_{i=1}^{n} \gamma^{t-1}r_{t}^{\left( i \right)}\rho_{t}^{\left( i \right)}}{\sum_{i=1}^{n} \rho_{t}^{\left( i \right)}};\rho_{t}\triangleq\Pi_{j=1}^{t}\frac{\pi_{\theta}\left( a_{j}|s_{j} \right)}{\pi_{T}\left( a_{j}|s_{j} \right)}$$

where $\pi_{\theta}$ and $\pi_{T}$ are learnt and policies. The two estimators are similarly constructed, where $n$ is number of trajectories in clinician’s behavioral trajectories $\mathcal{D}_{T}$ , and $T$ is number of steps in each trajectory. CWPDIS depends on number of trajectories retained when re-weighting $\rho$, the Effective Sample Size (ESS). A high ESS implies $\pi_{\theta}$ and $\pi_{T}$ have similar action distributions, thus reward received from $\pi_{T}$ could better estimate the value for $\pi_{\theta}$. In CWPDIS, $\rho_{t}$ is calculated for every step in a patient’ trajectory. For a deterministic policy, $\rho_{t}$ will return true if actions from $\pi_{\theta}$ are the same from $\pi_{T}$ for every single step in a trajectory, whereas $\rho_{t}$ will continue to return true in each step until $\pi_{\theta}$ predicts a different action from $\pi_{T}$ . CWPDIS evaluates quality of learnt policies considering ESS $\rho_{t}$ and reward in each step, where $\rho_{t}$ measures consistency between clinician’s policy and learnt policies.

1. **EXPERIMENTAL RESULTS**

In this section, we illustrate the mortality vs. expected return for sepsis cohort and HbA1c-High rate vs. expected return for diabetic cohorts, respectively.

### Mortality vs. Expected Return for Sepsis Cohort

Figure 1 illustrates changes in mortality against the differences between clinician and corresponding policy recommended dosages for sepsis.

### HbA1c-High Rate vs. Expected Return for Diabetic Cohort

Figure 2 illustrates changes in HbA1C-High rates against the differences between clinician and corresponding policy recommended dosages for diabetes.

### Algorithmic Convergence

Experimental evidence clearly demonstrates convergence of the proposed policy learning process. We plot training loss values for policy learning in phase 2 for sepsis and diabetes (see Figure 3). Loss values for SI-S1, SI-S2 and SI-D steadily decrease over time and eventually stabilize, indicating models are effectively learning.


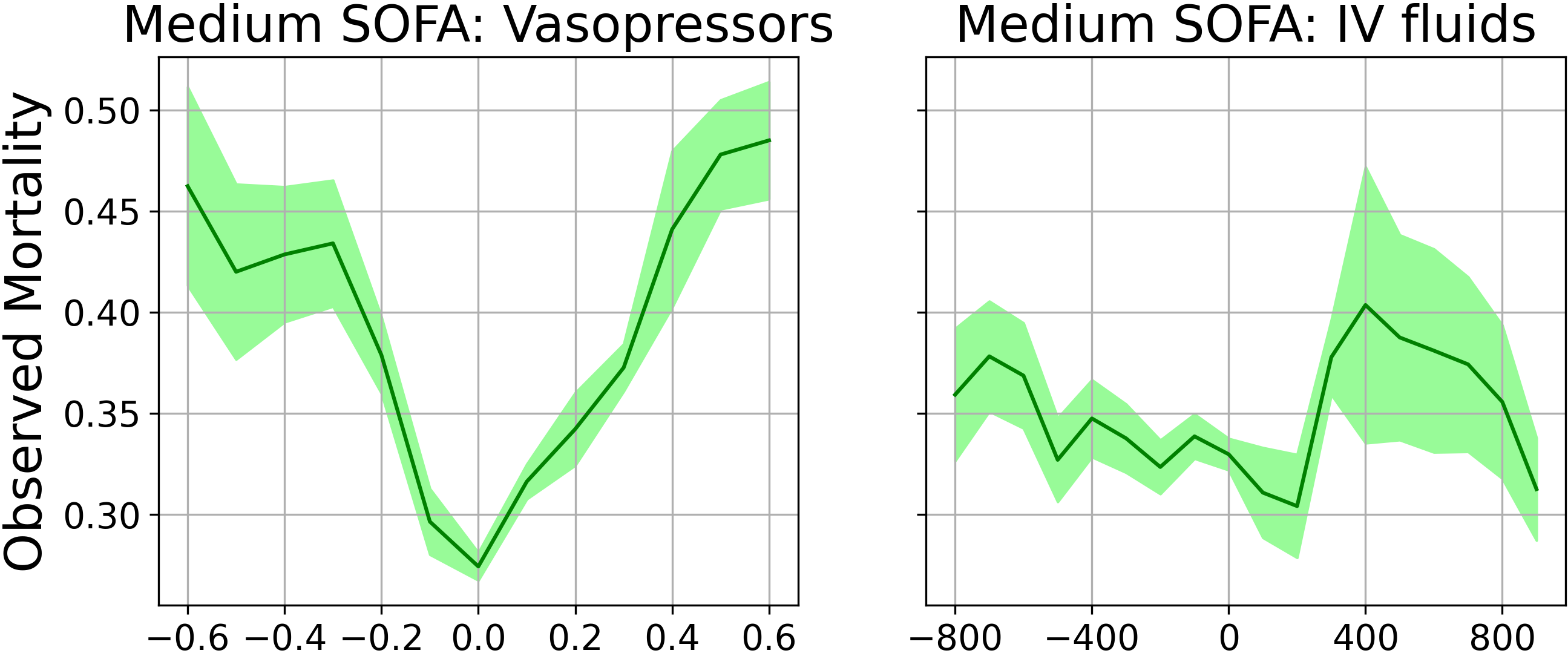

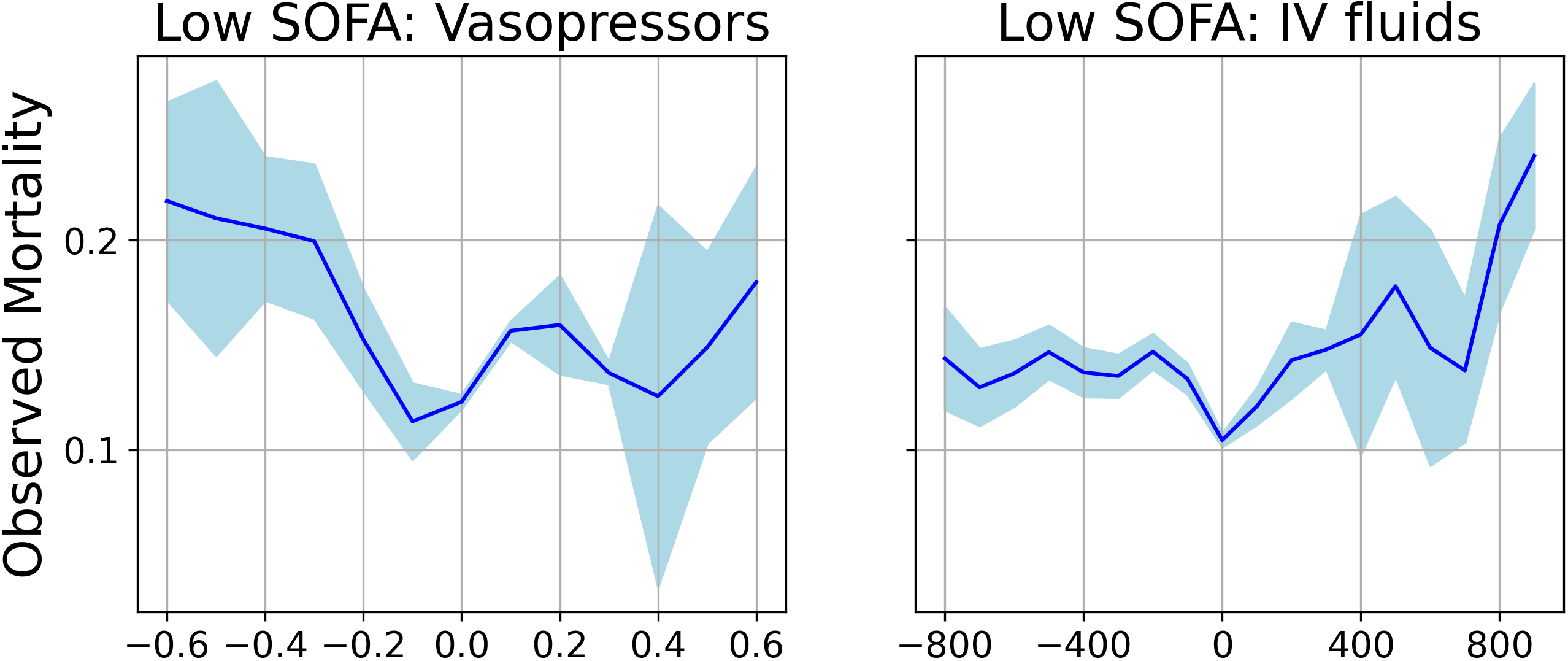

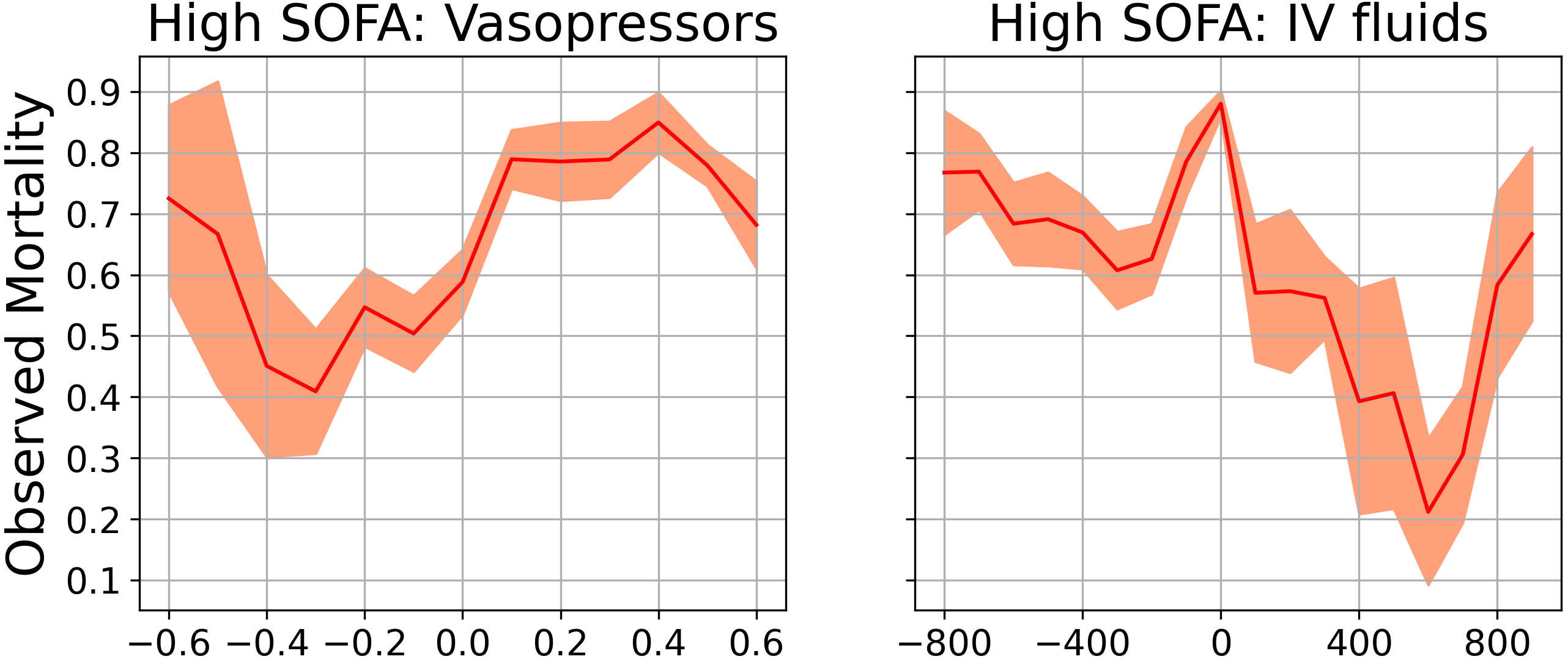


NFQ


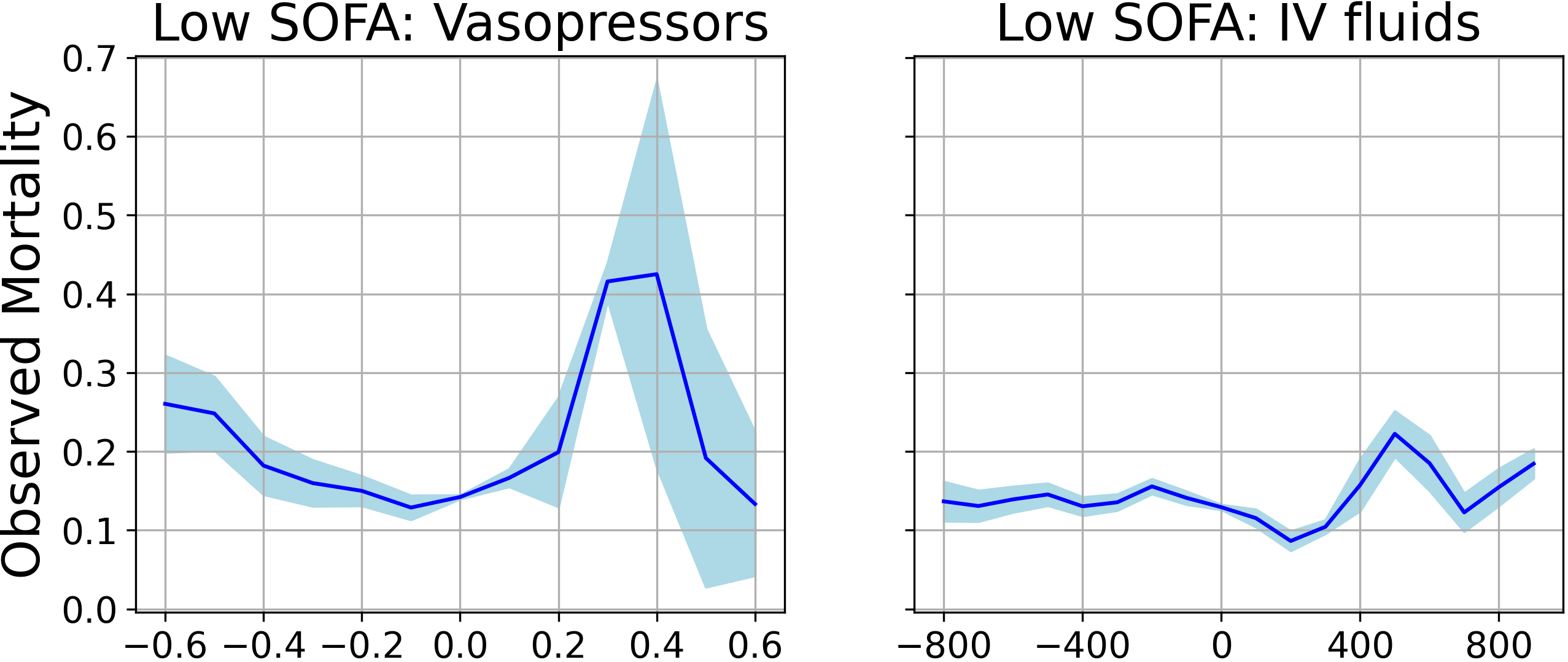

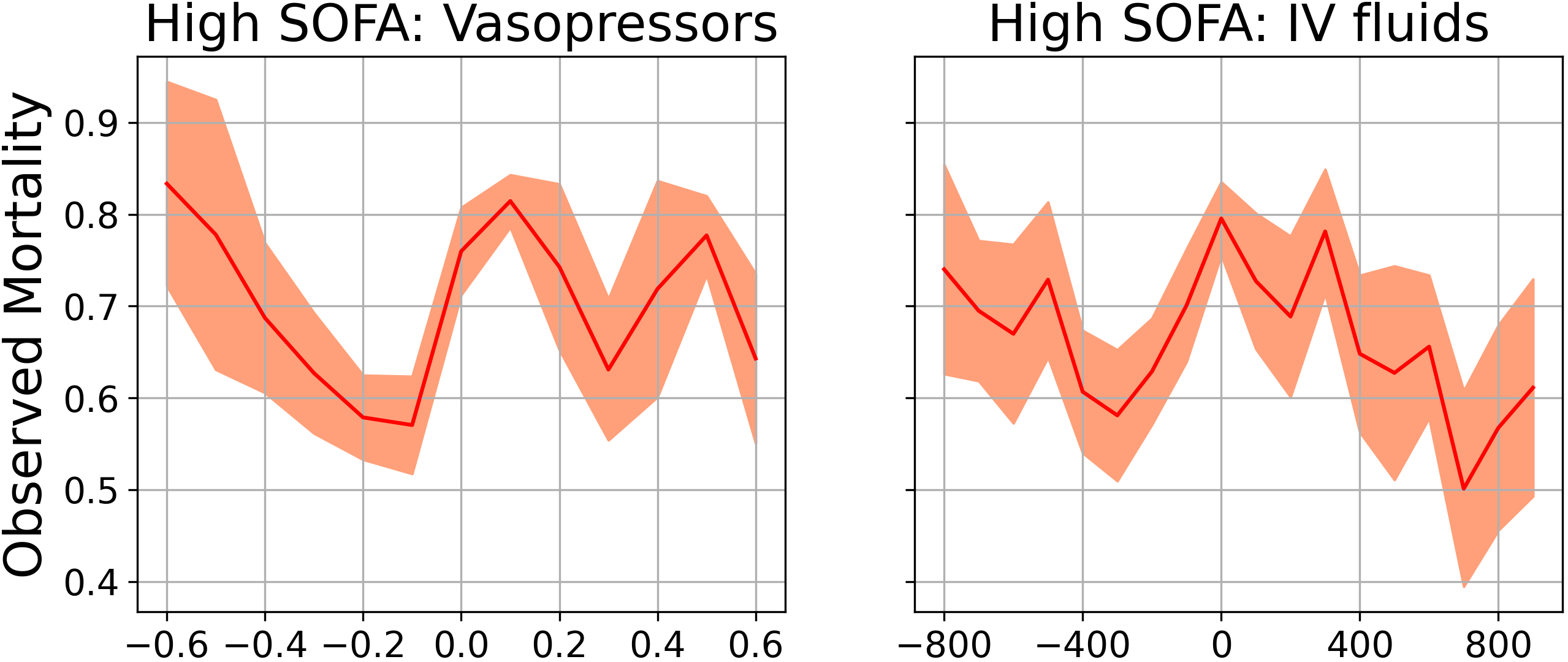

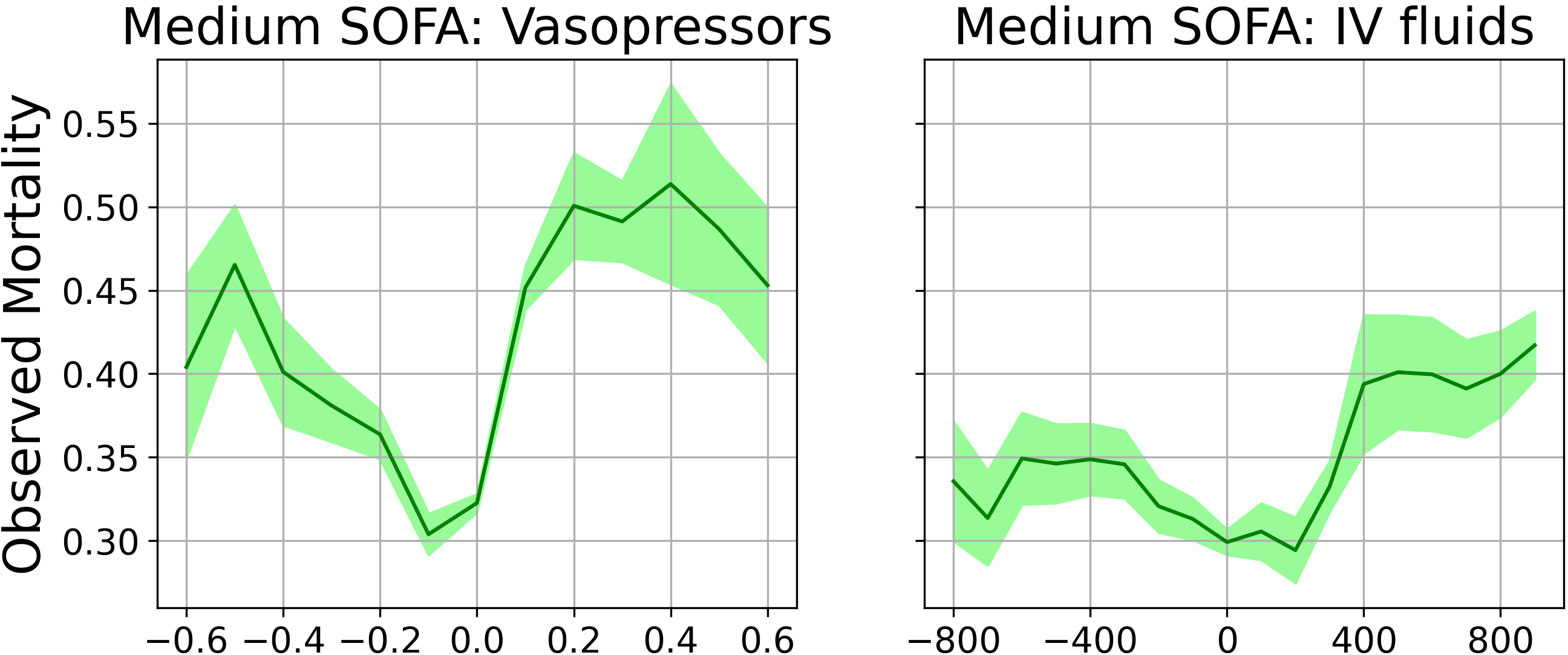


D3QN


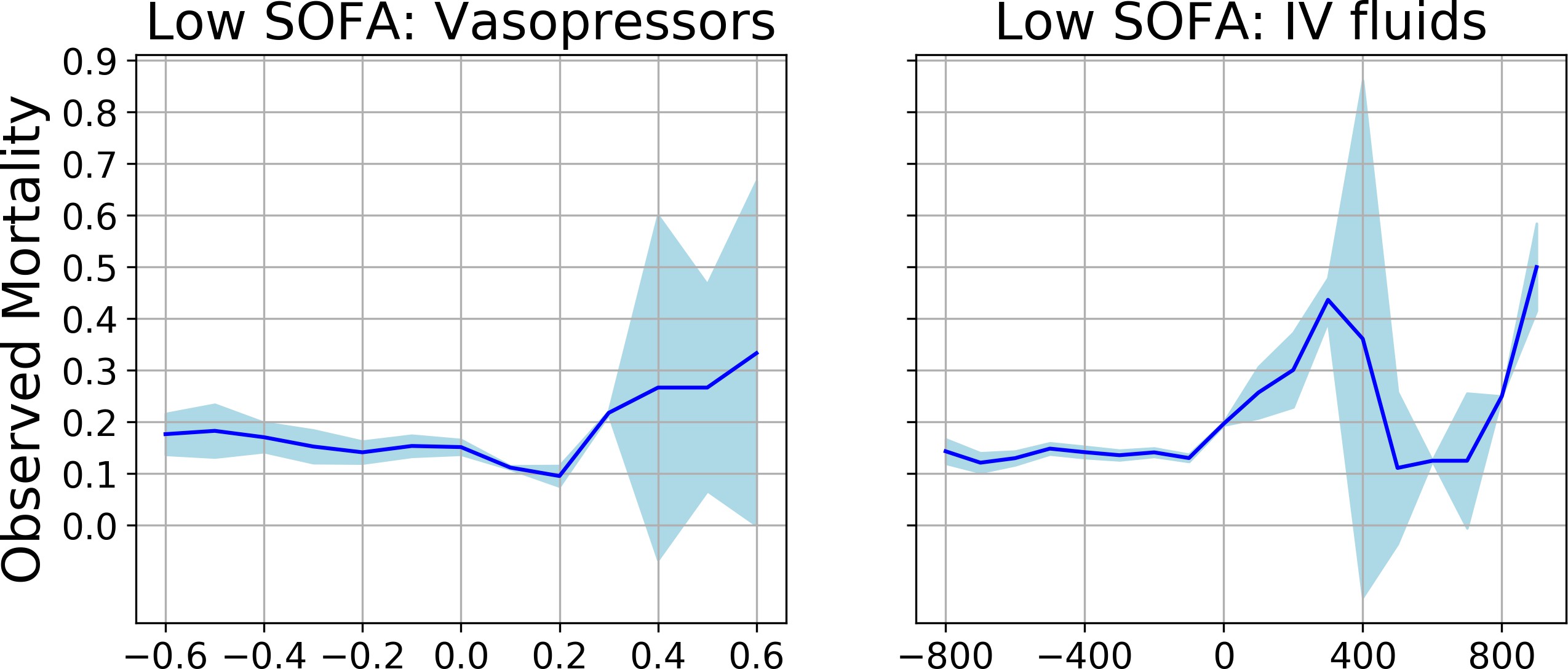

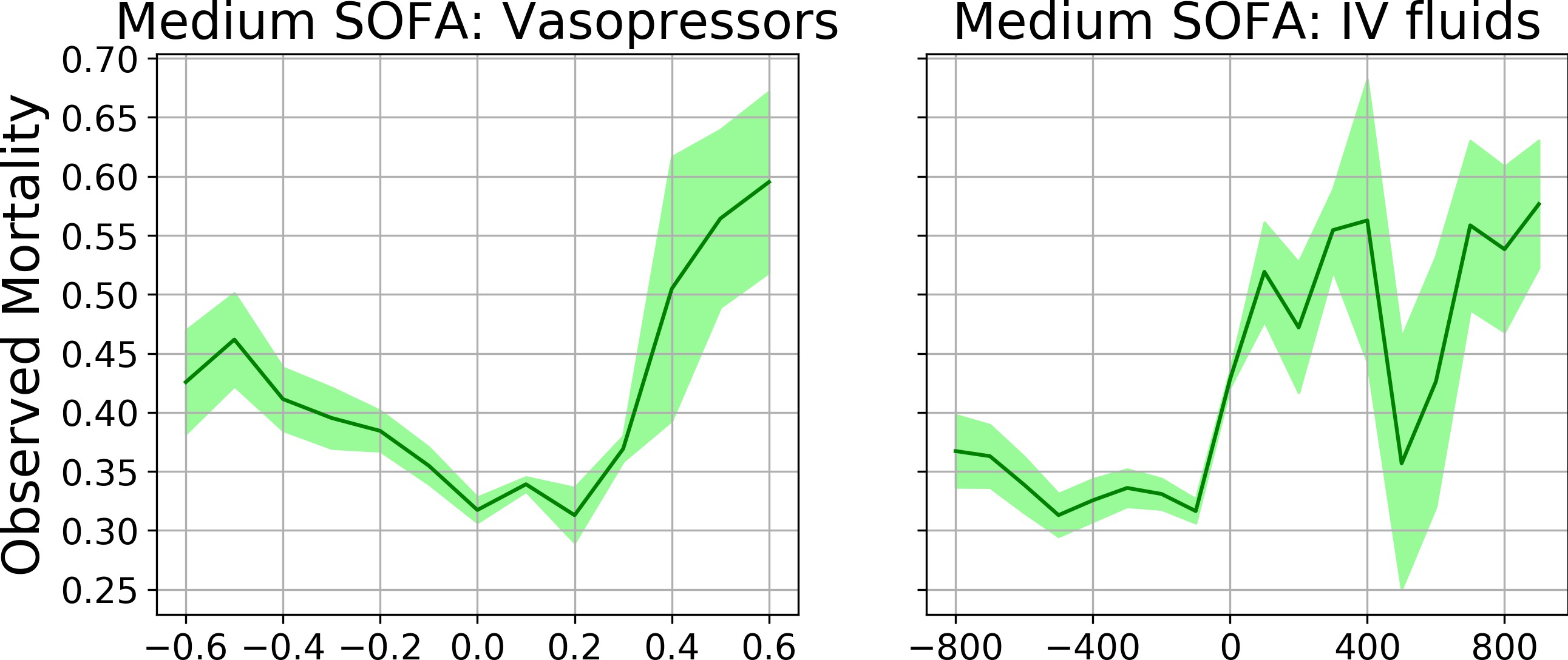

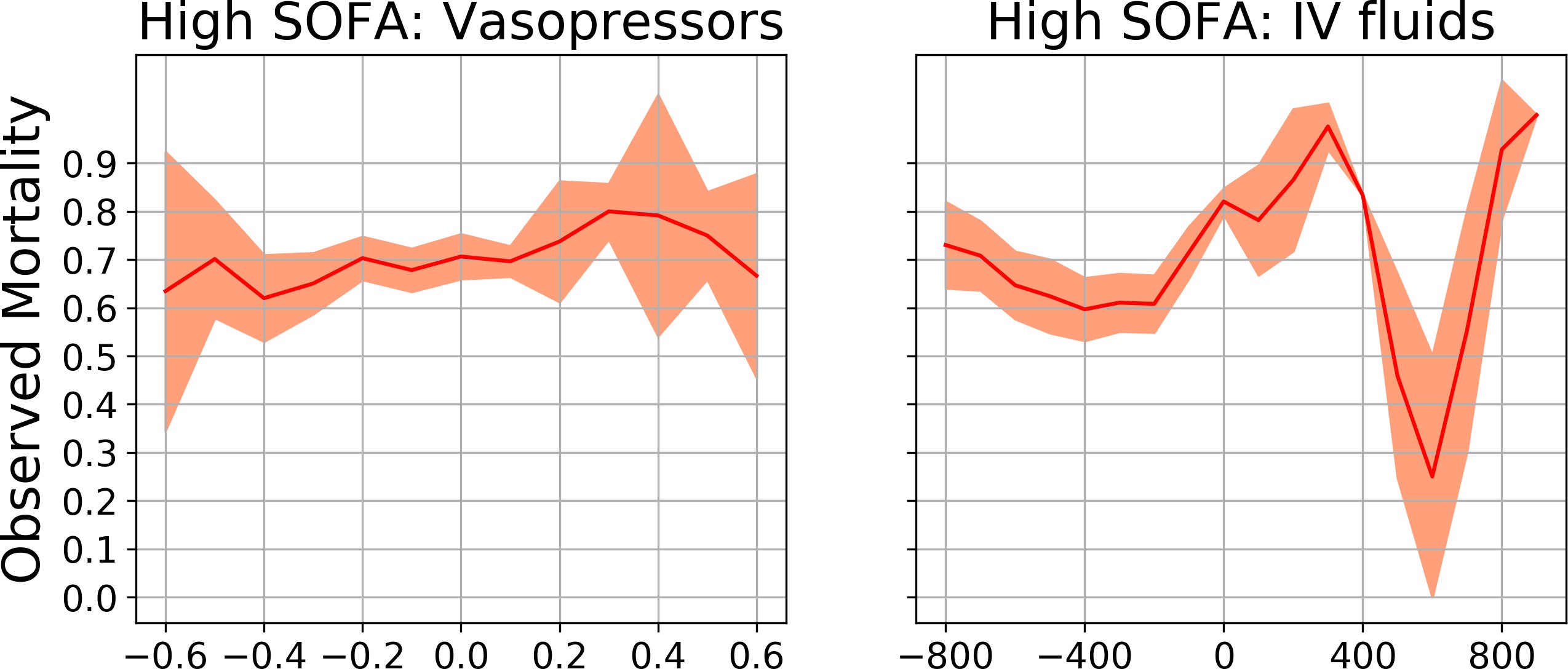


POfD


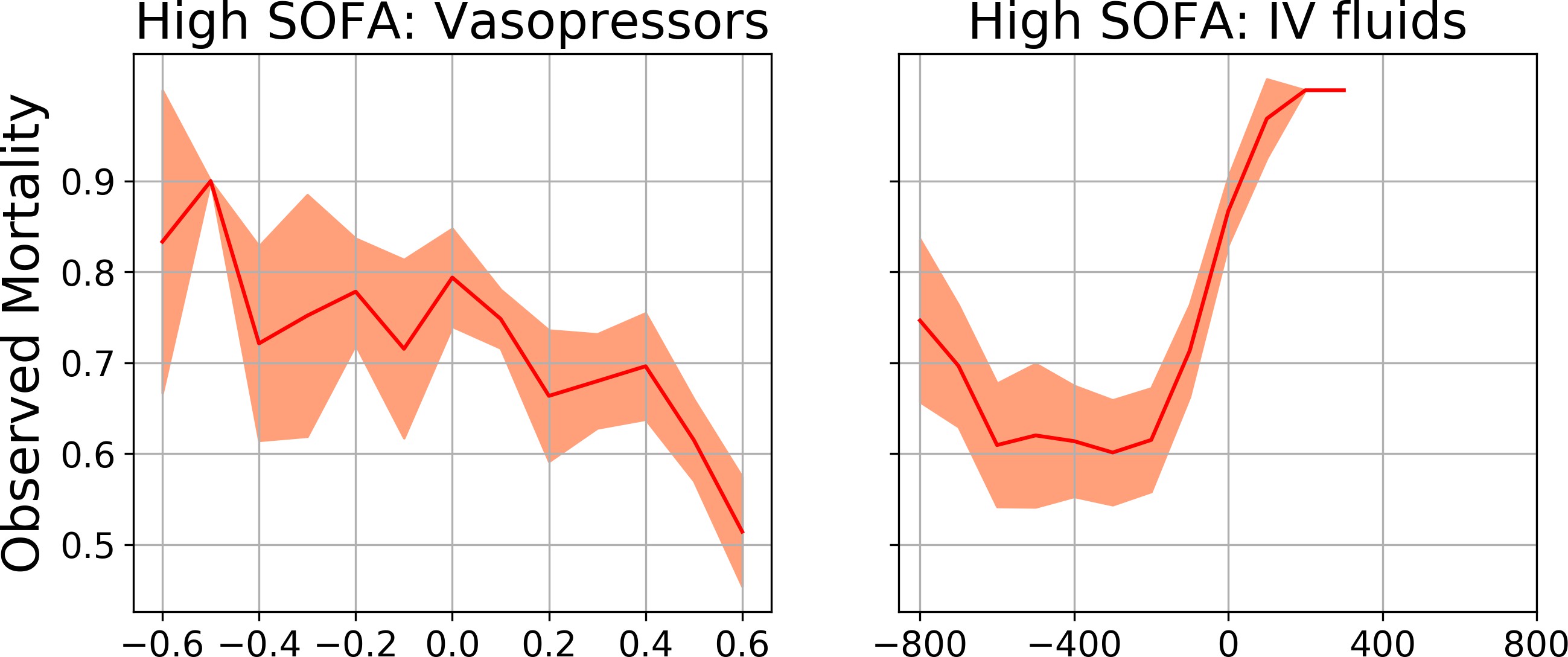

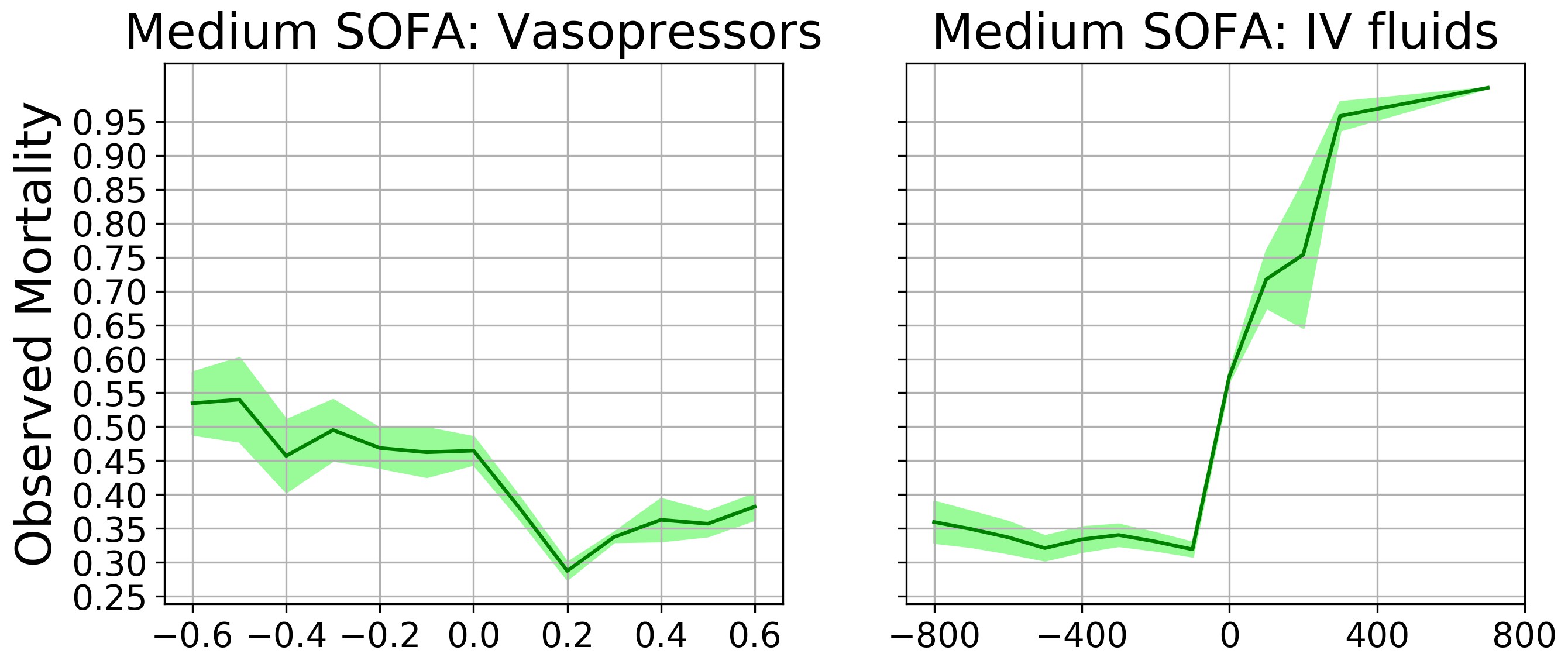

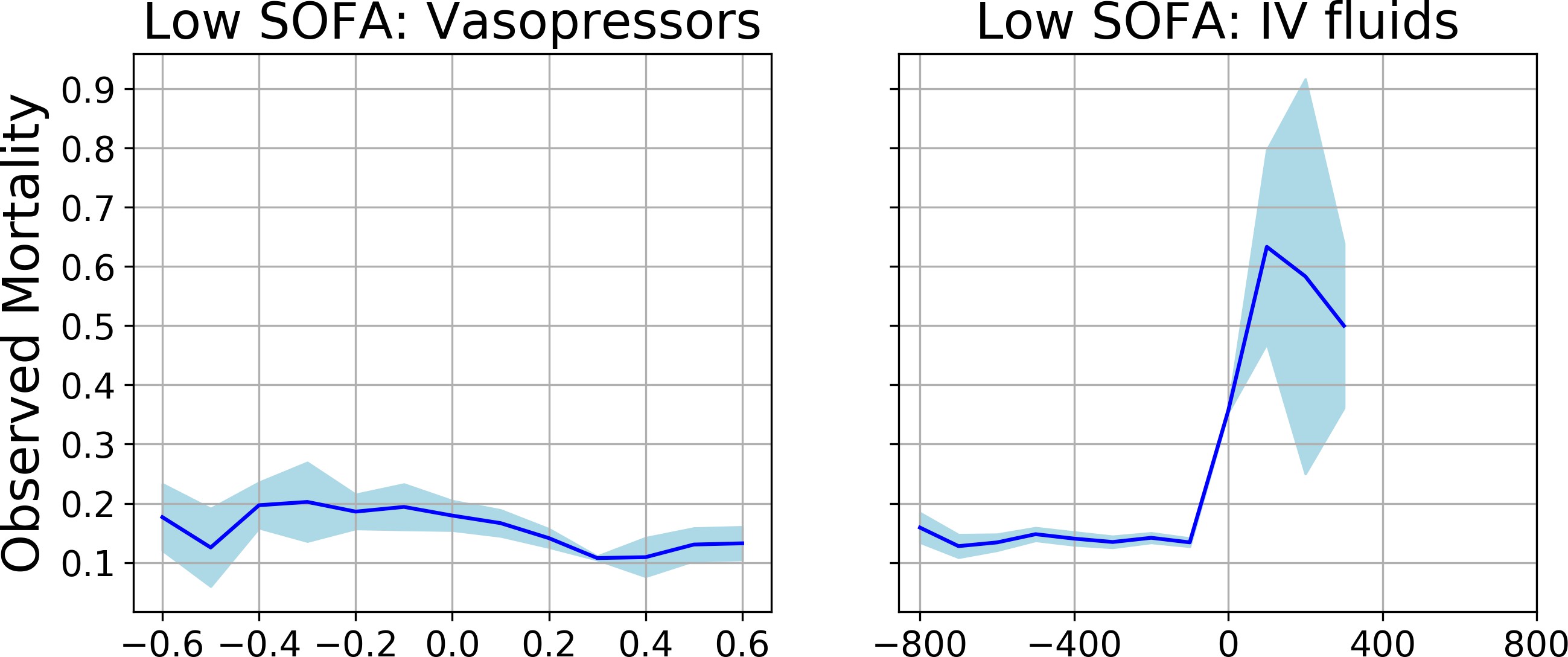


IC-GAIL


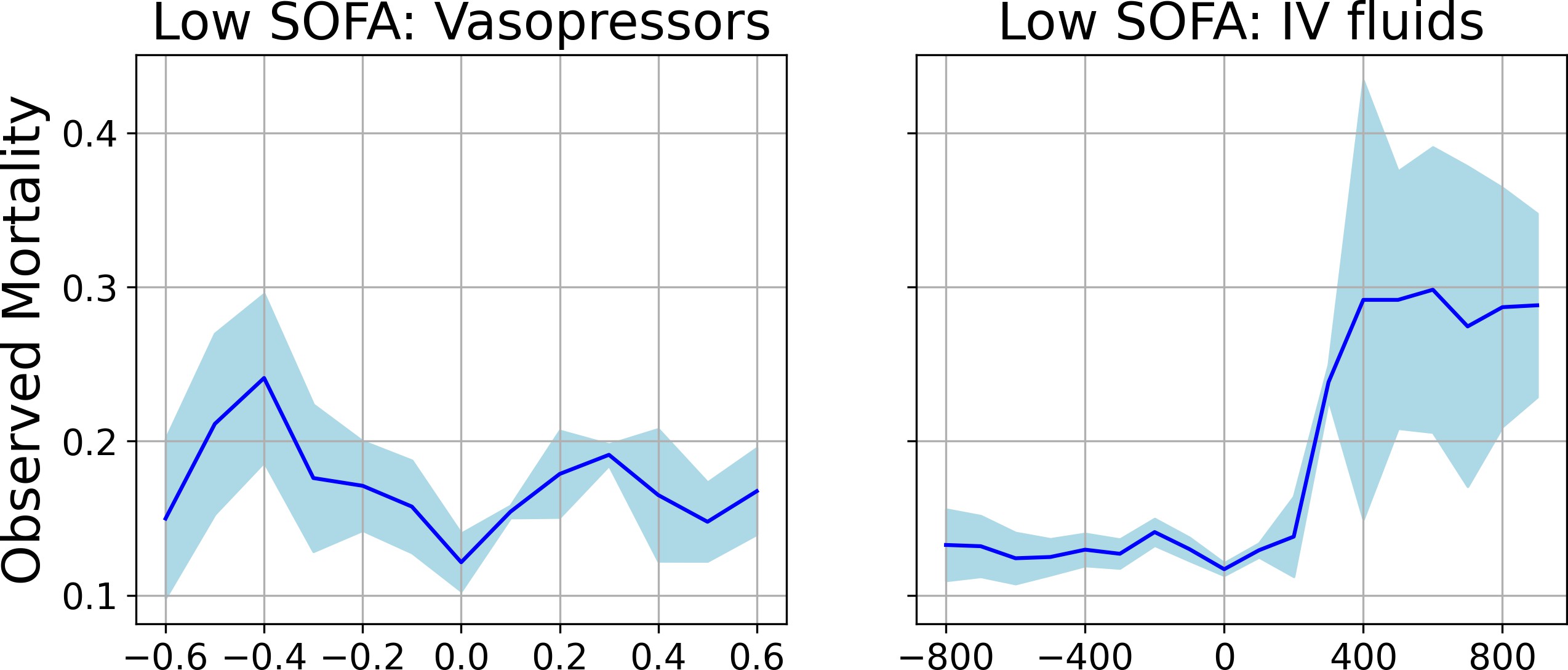

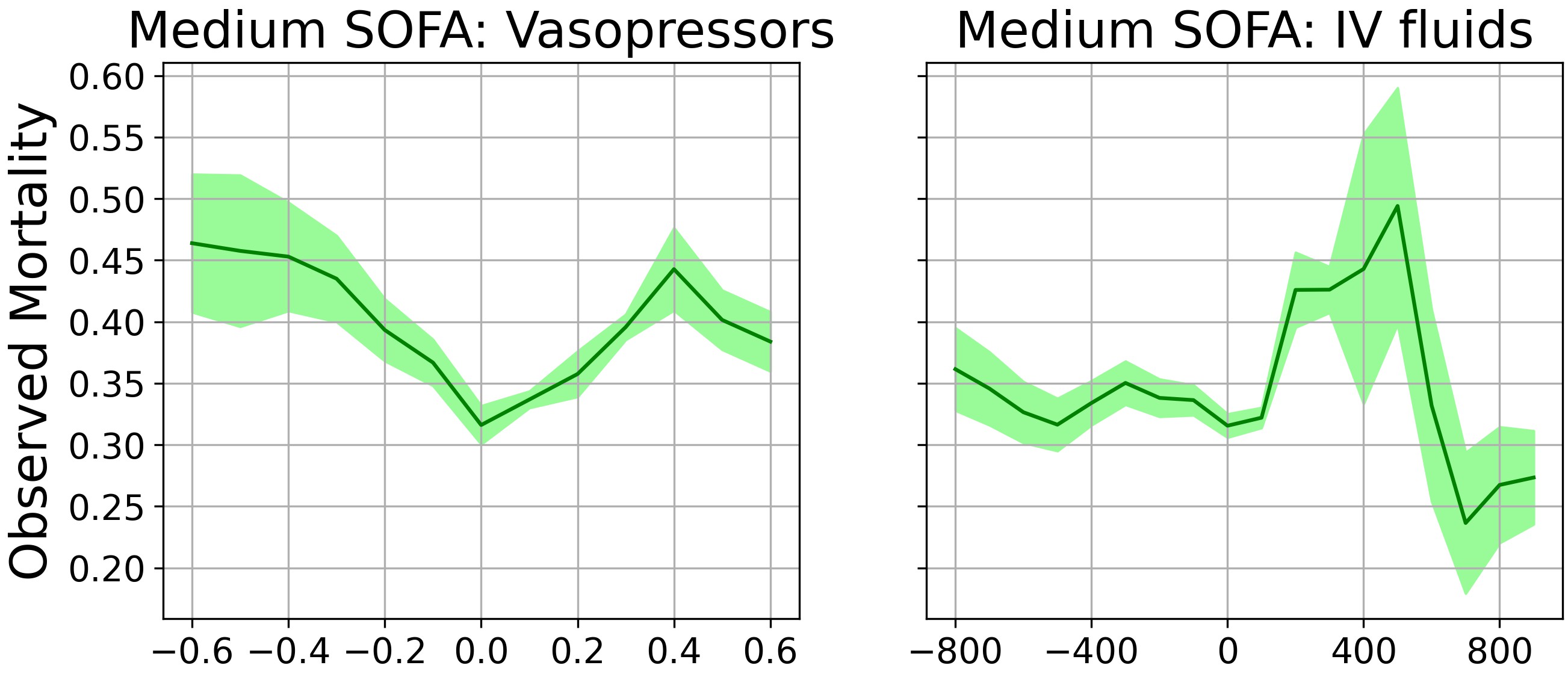

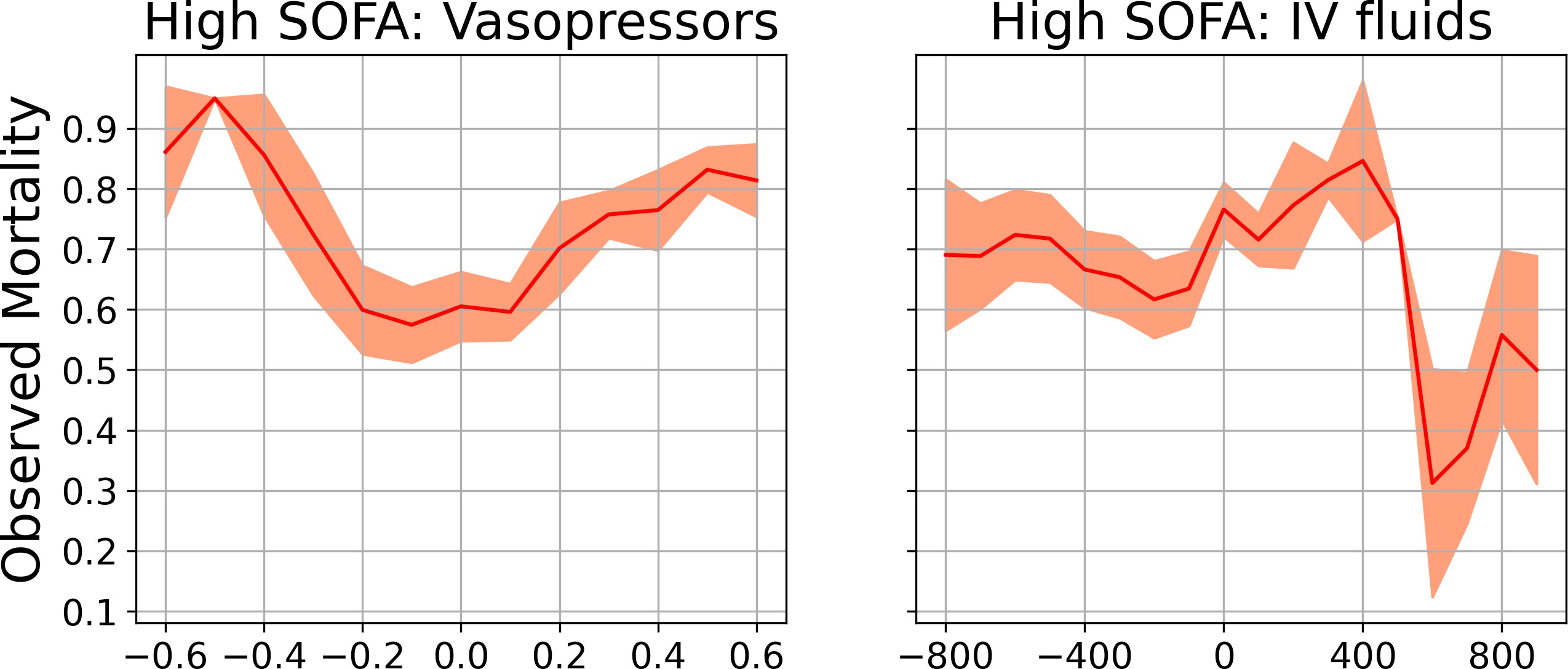


ACIL


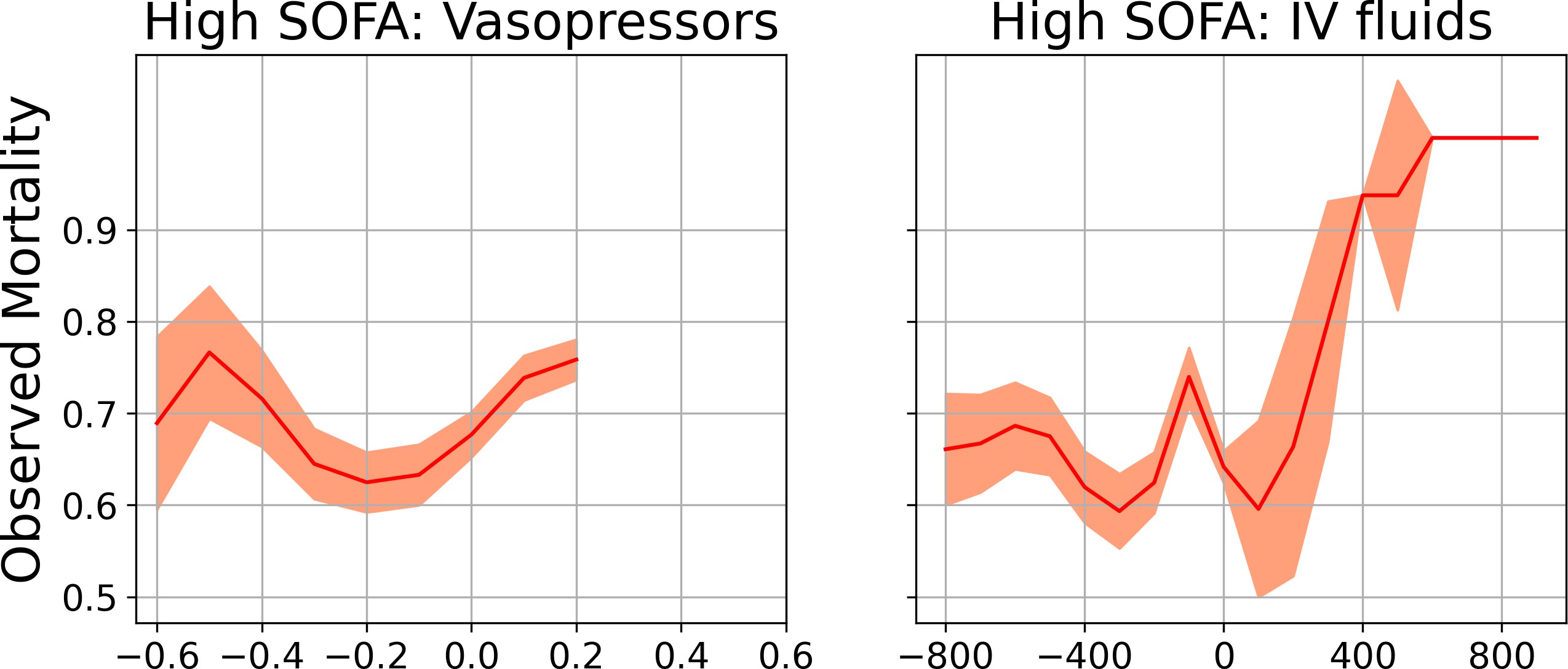

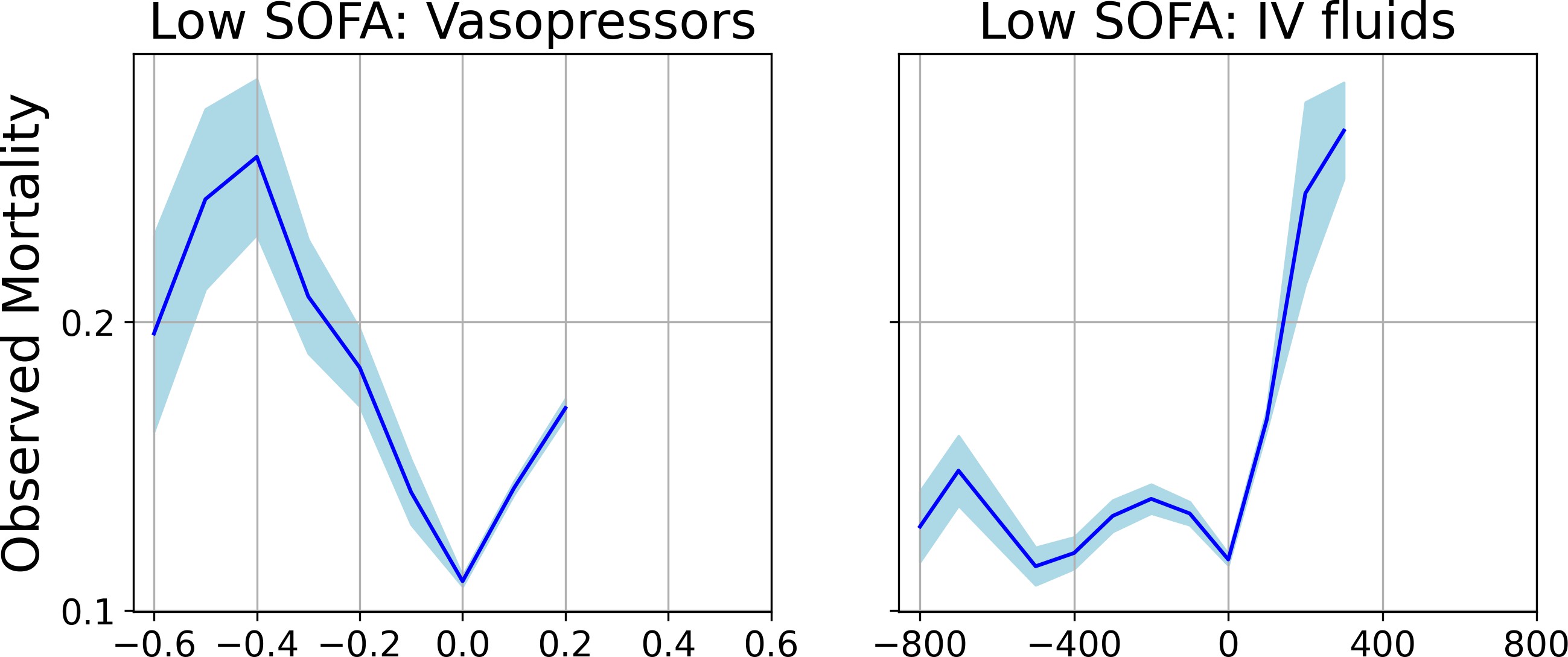

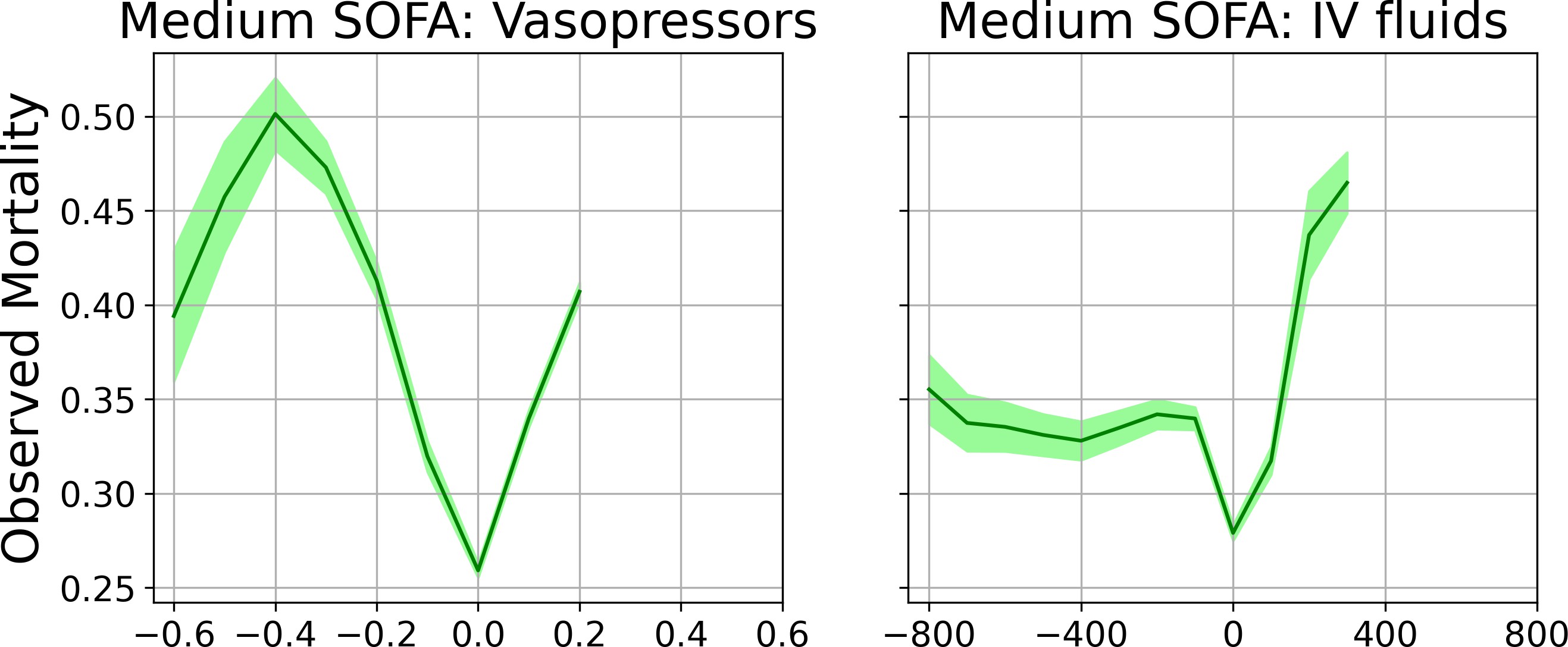


SI-S1


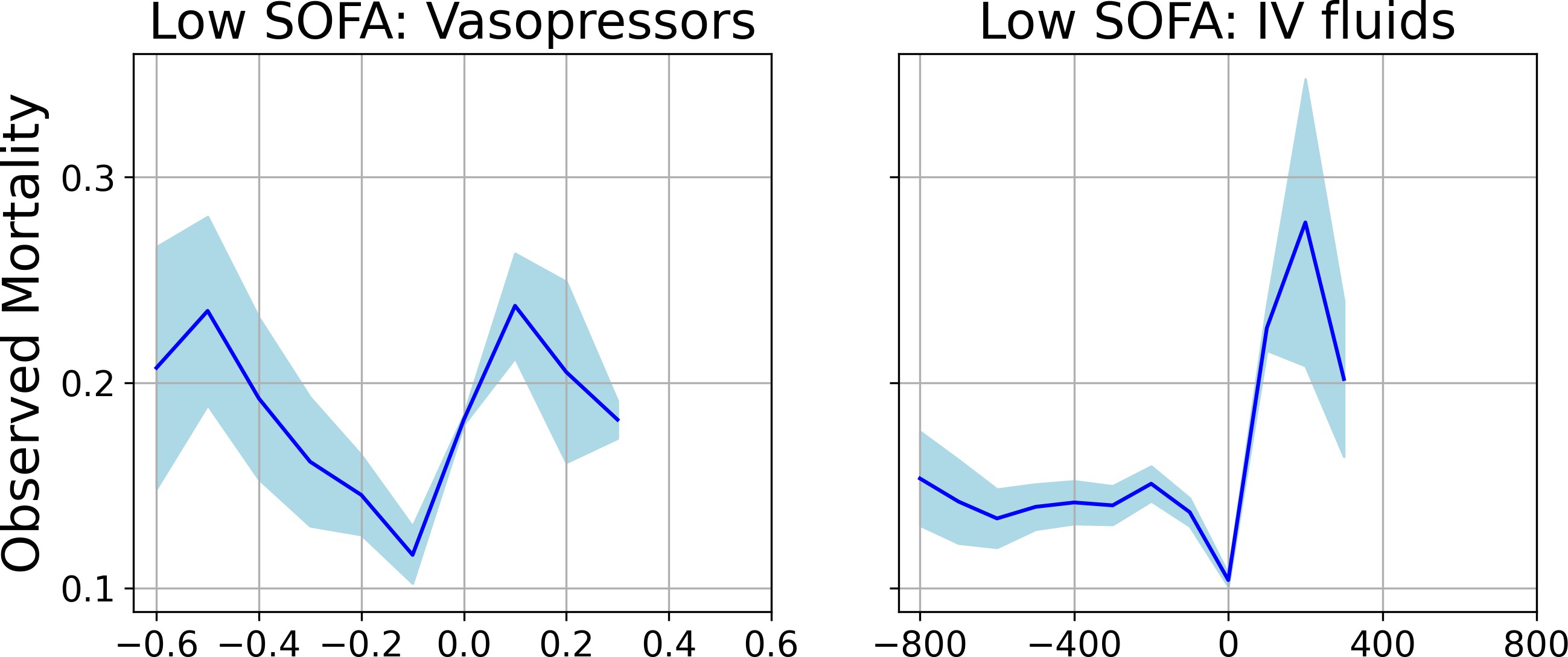

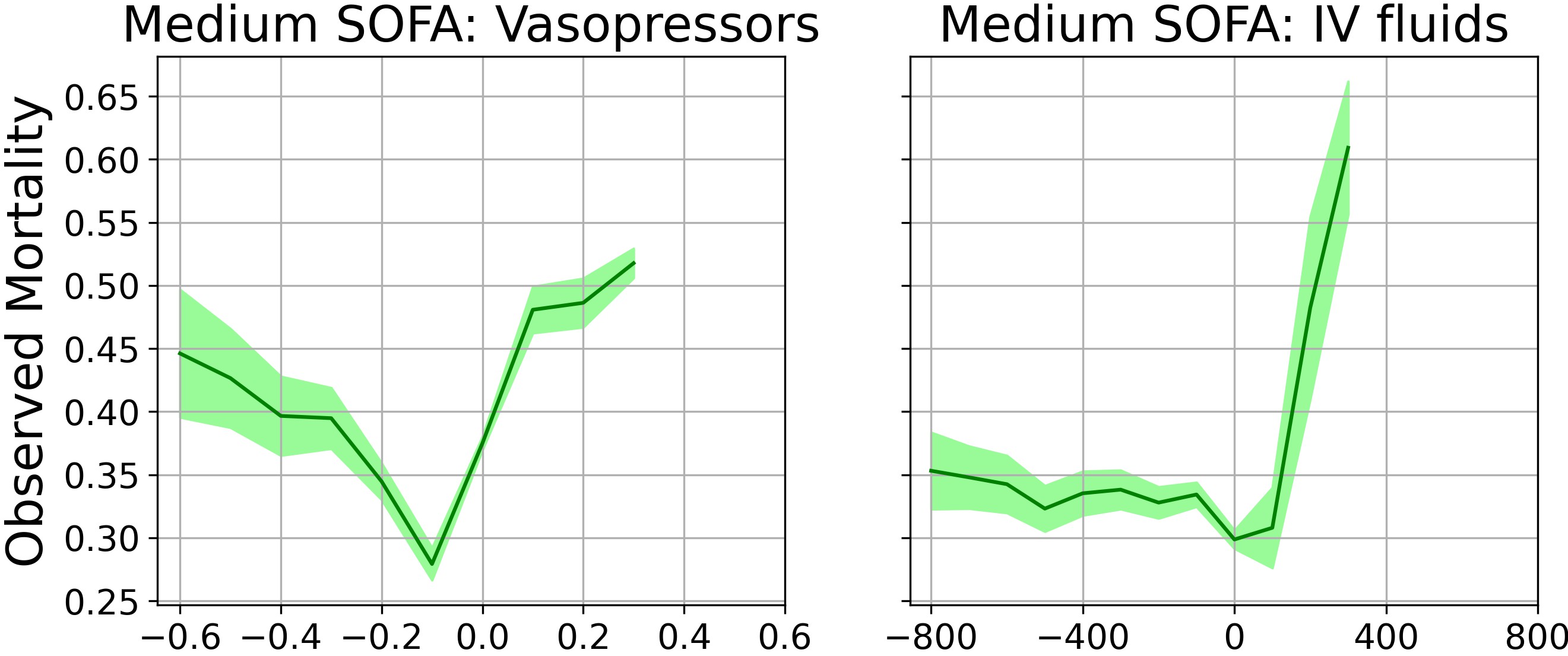

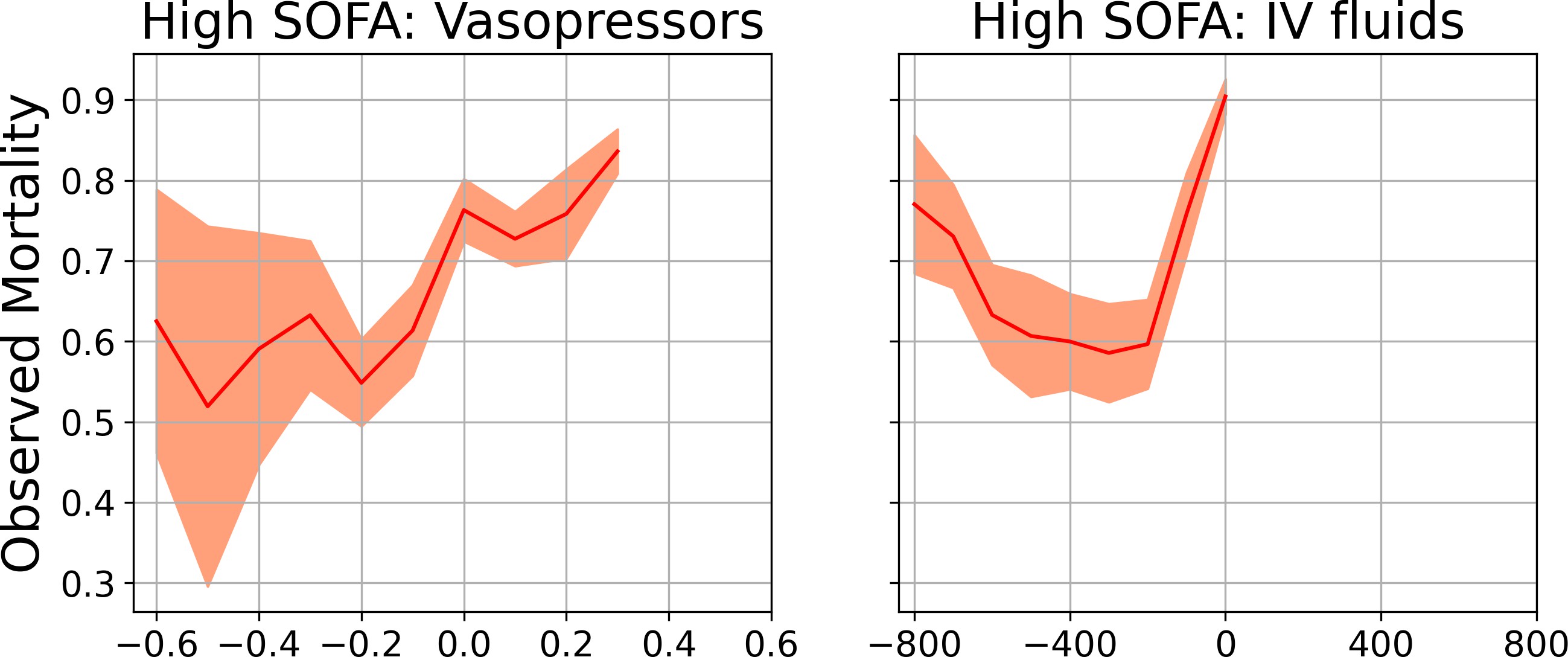


SI-S2

Figure 1: Changes in observed mortality (y-axis) versus the difference between the dosages recommended by the optimal policy and the dosages administered by clinicians (x-axis) on a held-out test set for the **Sepsis use case**. Graphs indicate the results for timesteps with Low (blue), Medium (green) and High (red) SOFA scores for all the models.


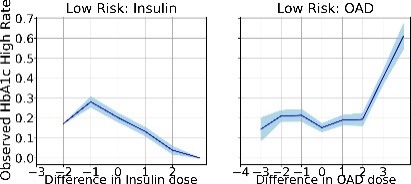

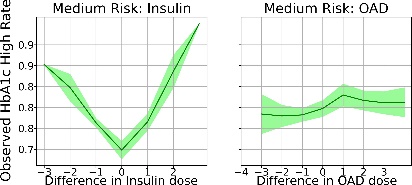

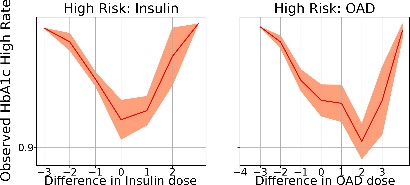


NFQ


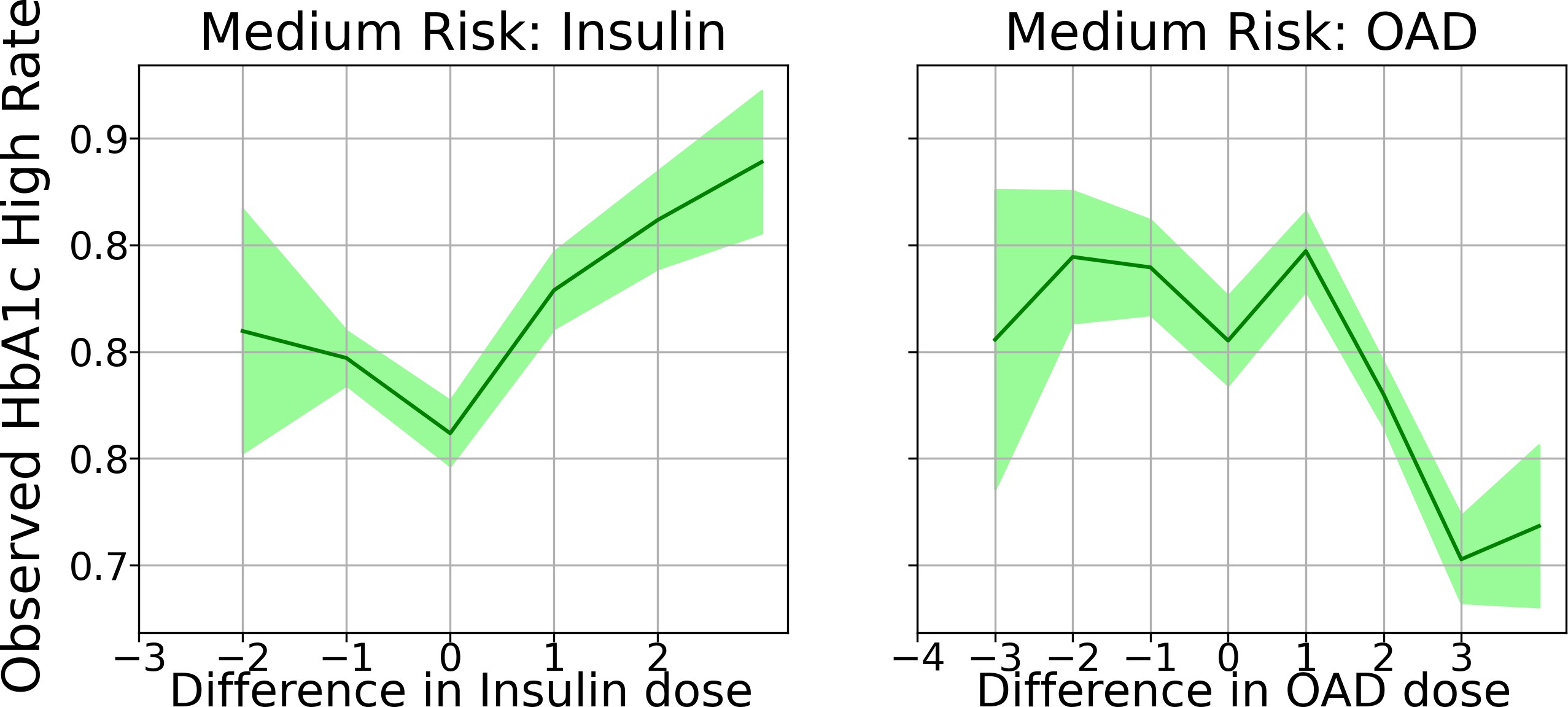

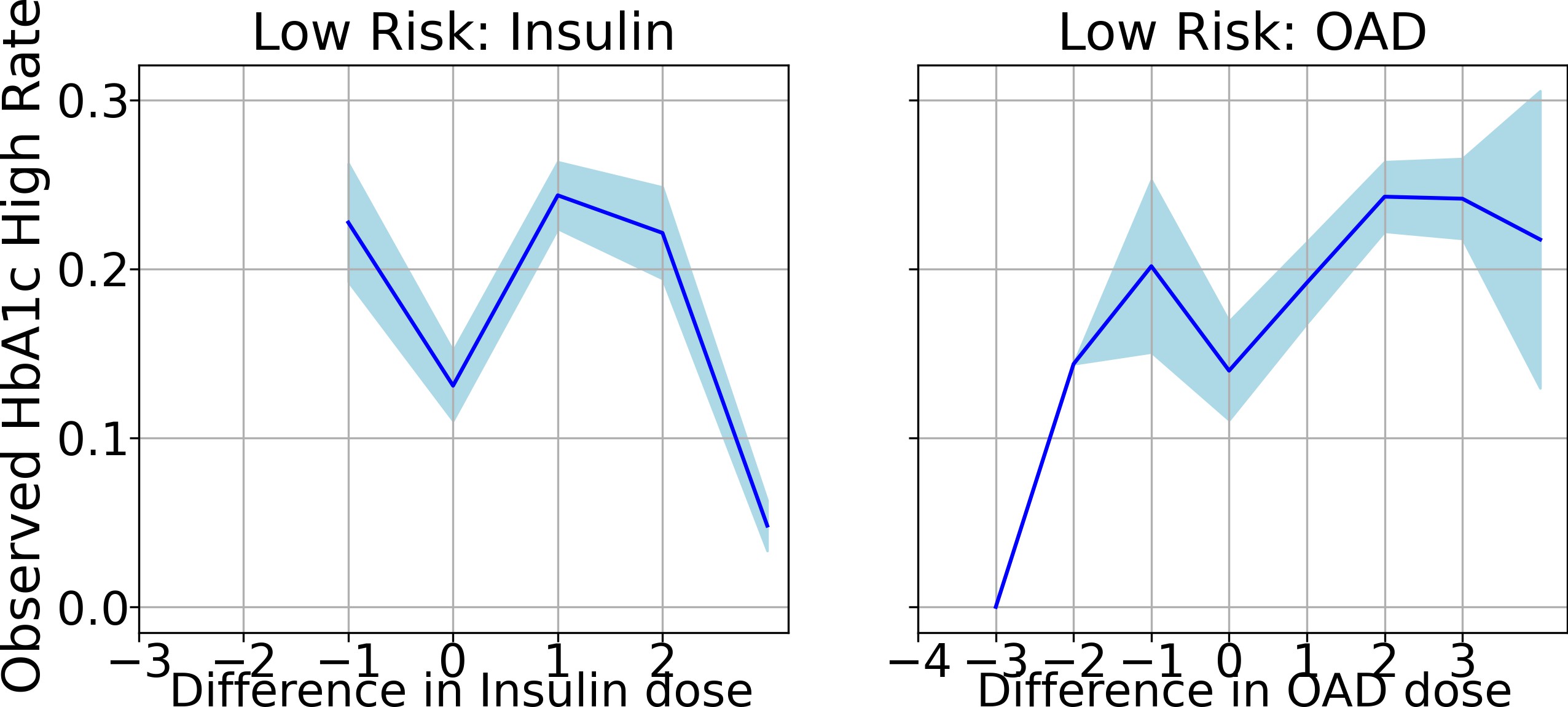

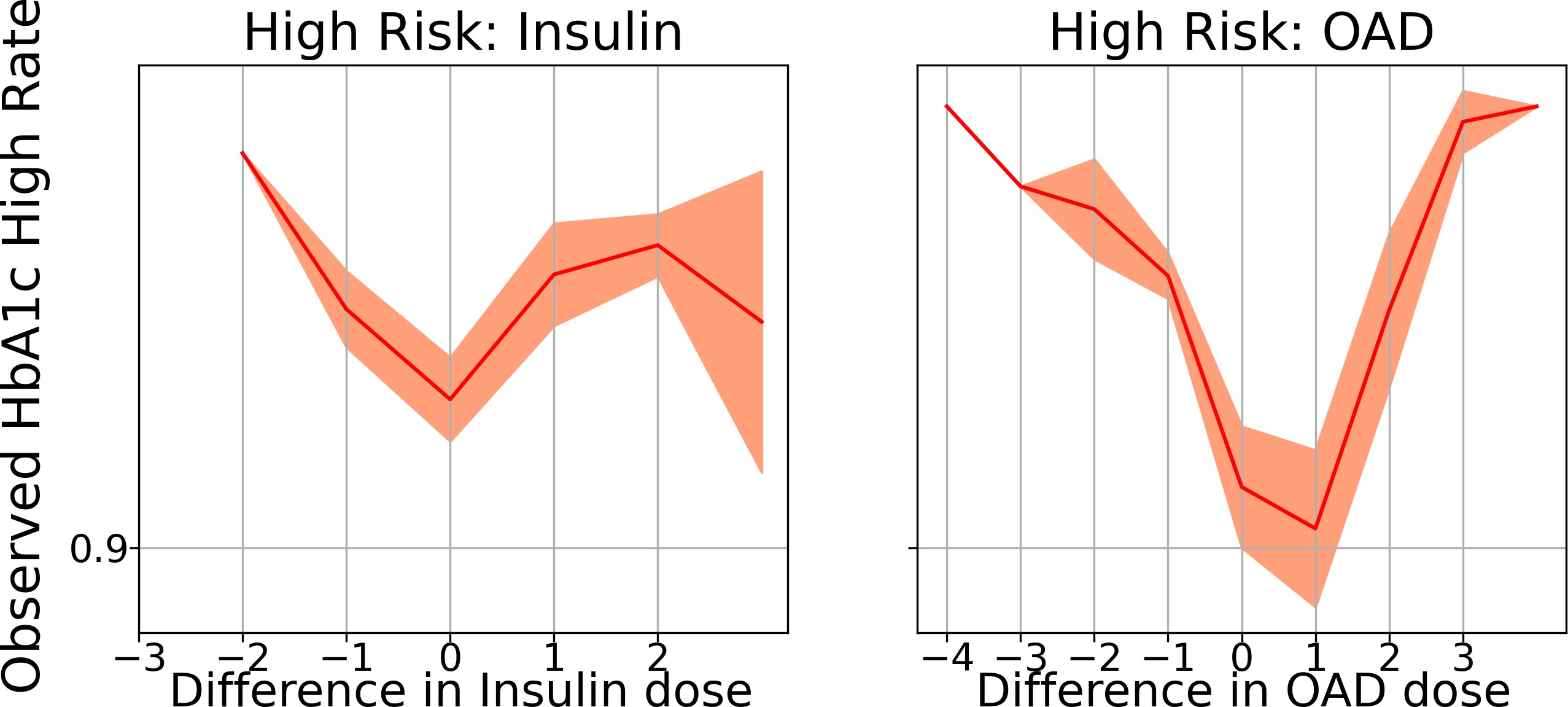


D3QN


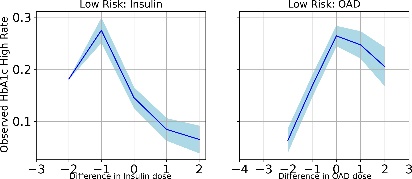

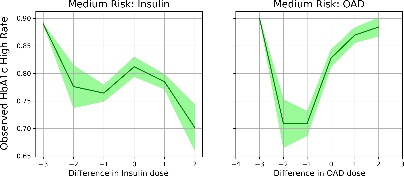

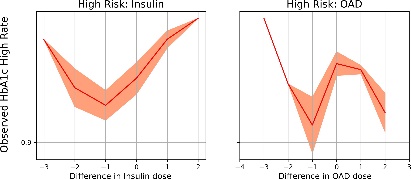


POfD


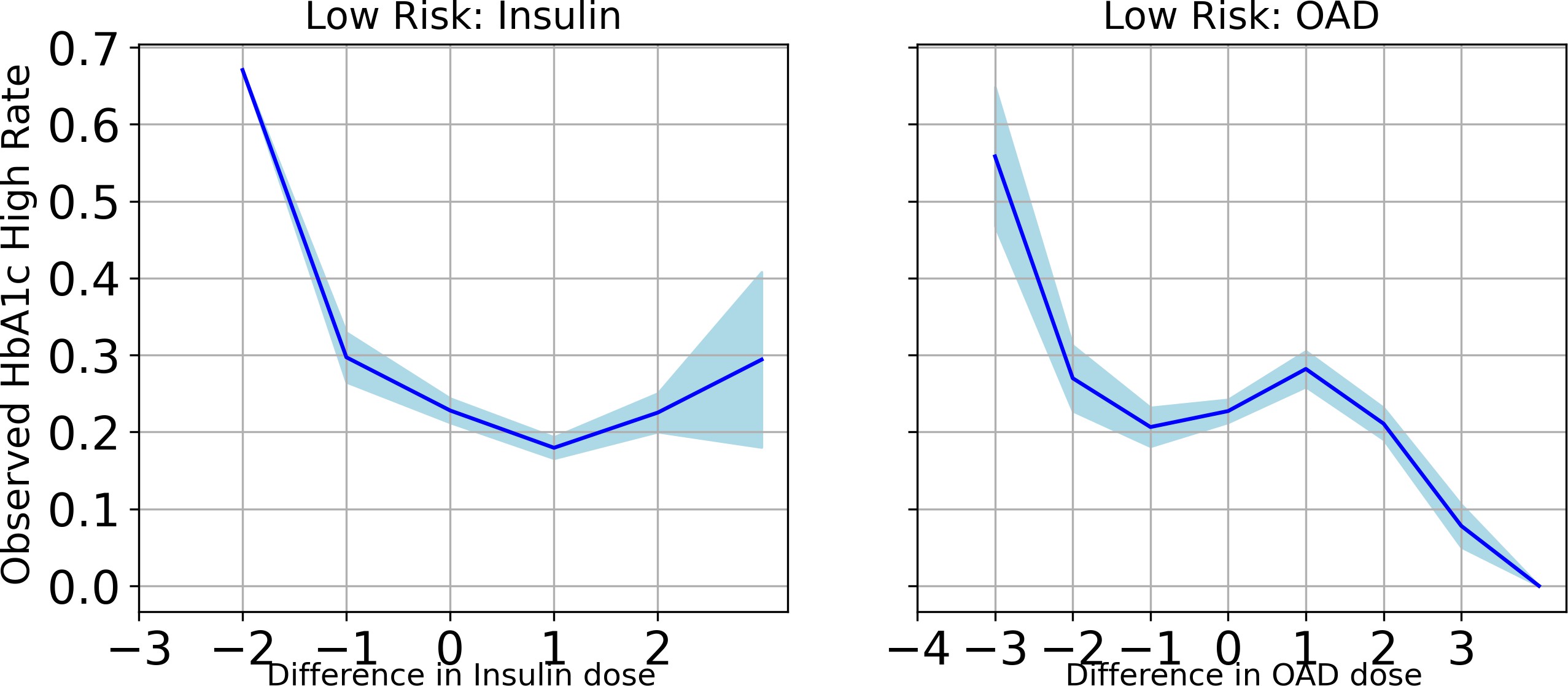

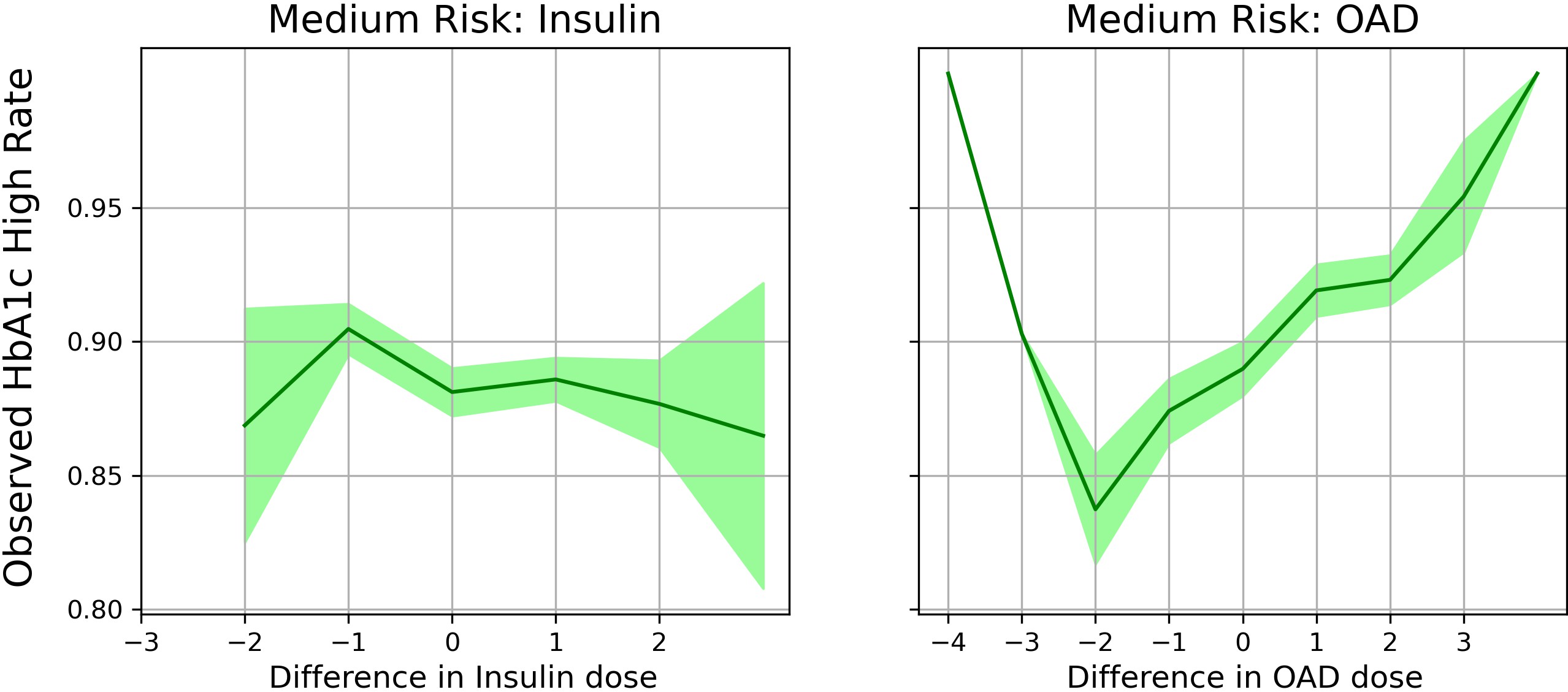

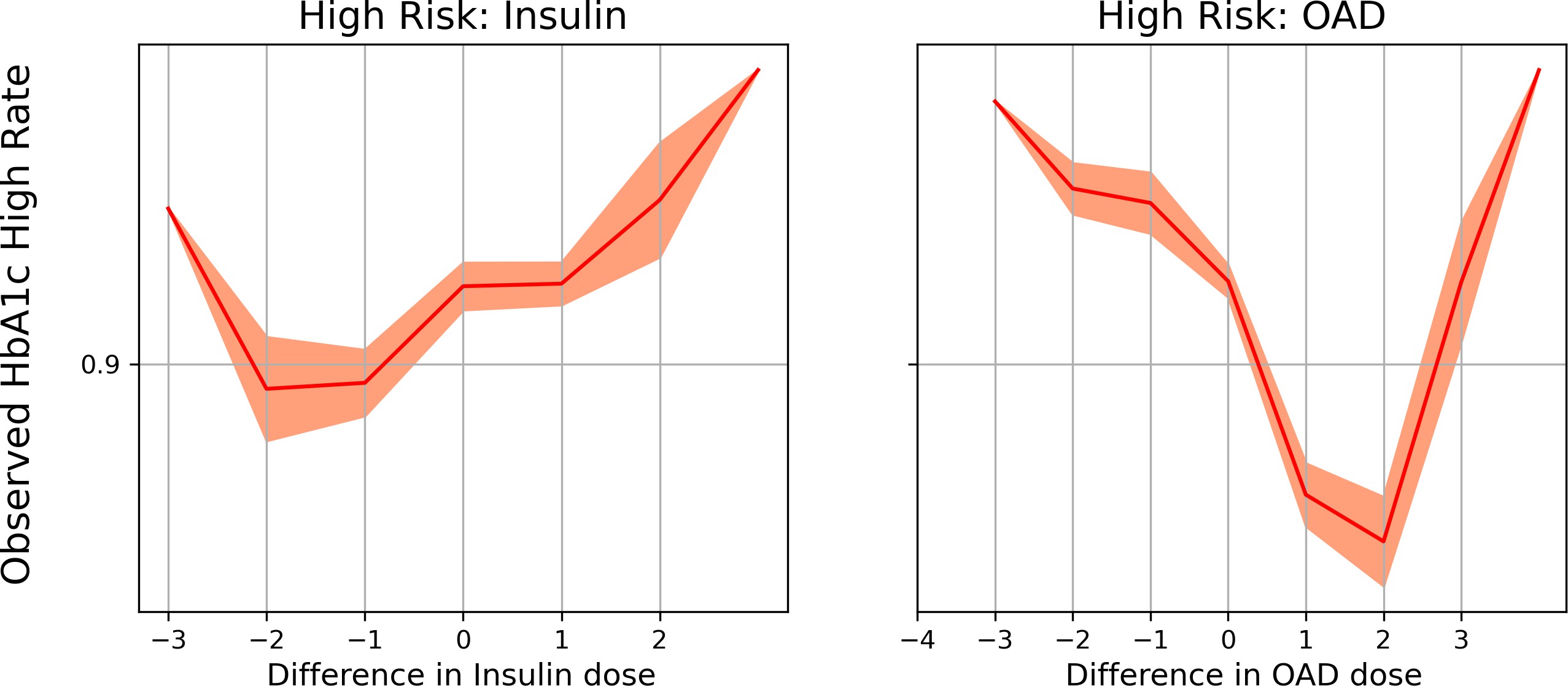


IC-GAIL


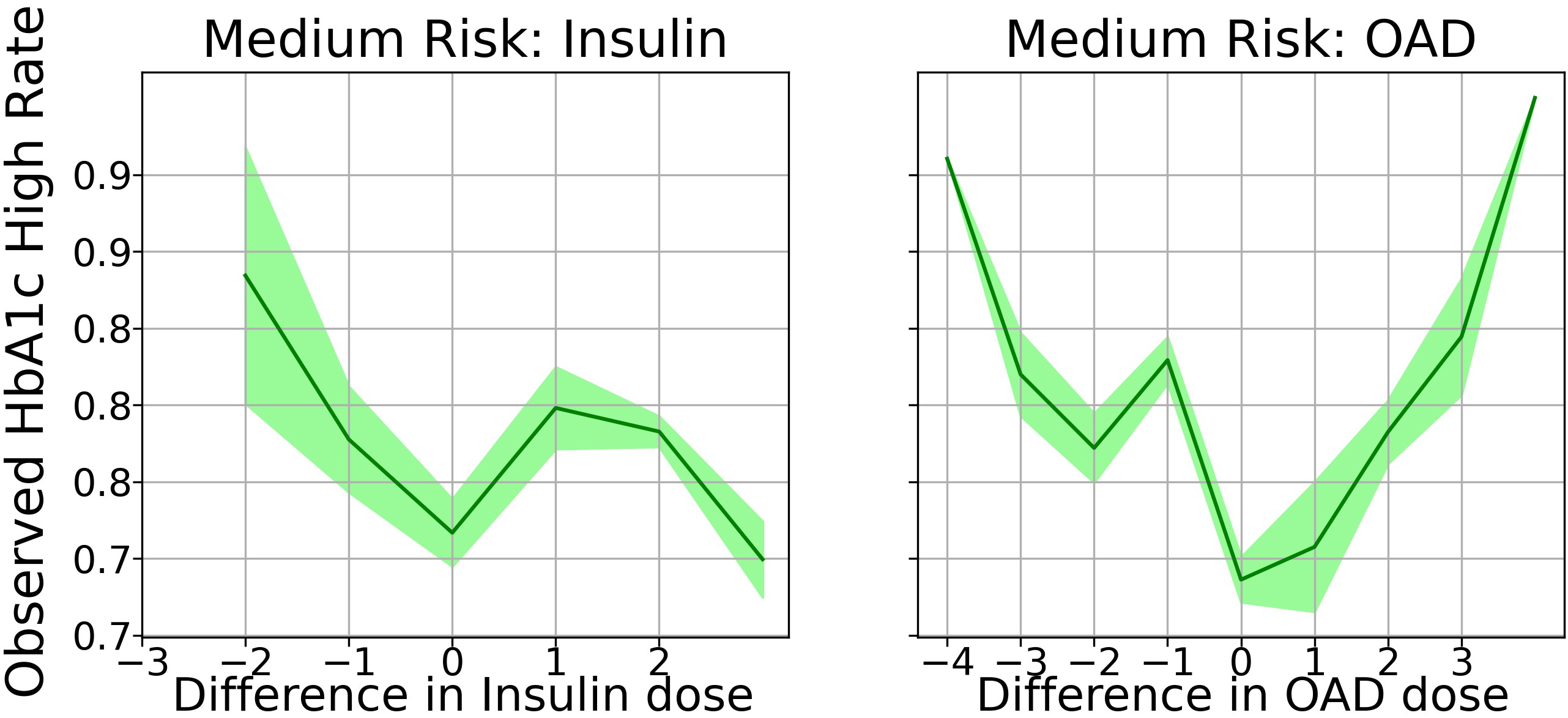

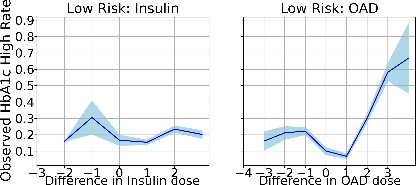

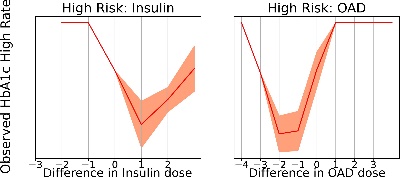


ACIL


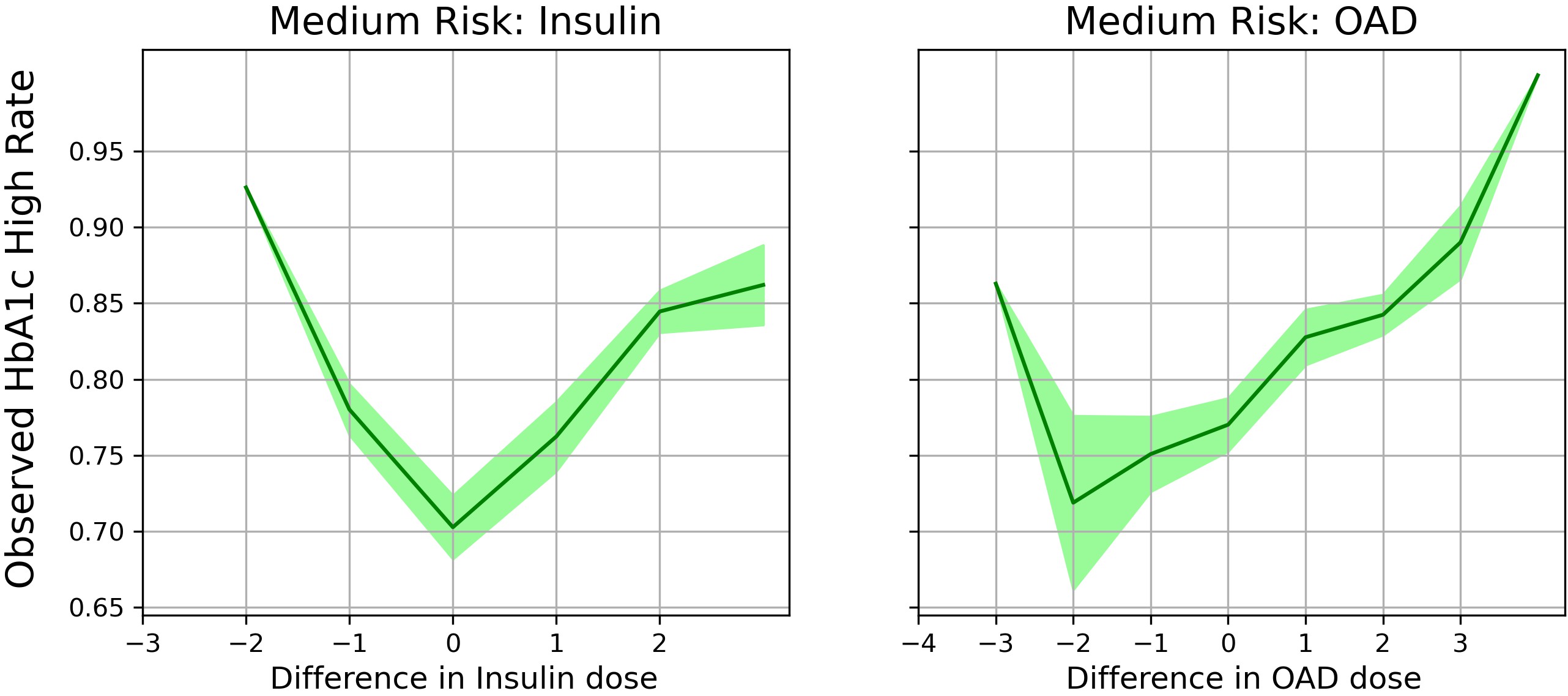

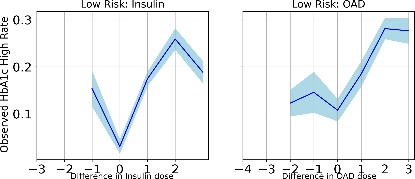

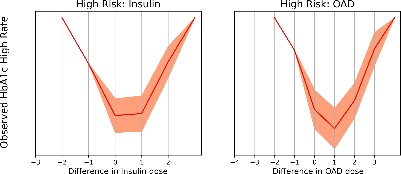


SI-D

Figure 2: Changes in observed mortality (y-axis) versus the difference between the dosages recommended by the optimal policy and the dosages administered by clinicians (x-axis) on a held-out test set for the **Diabetes use case**. Graphs indicate the results for timesteps with Low (blue), Medium (green) and High (red) HbA1C scores for all the models.


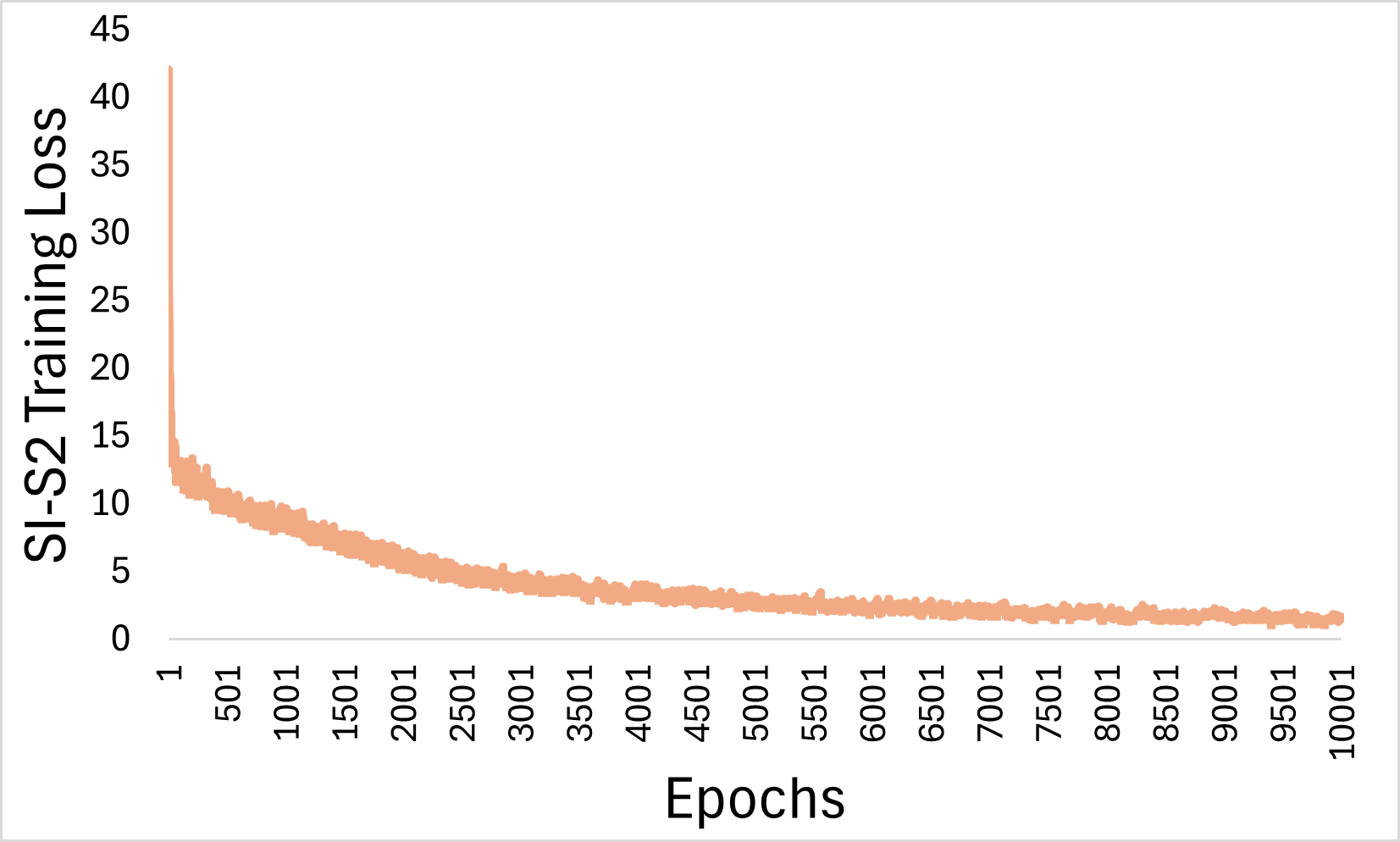

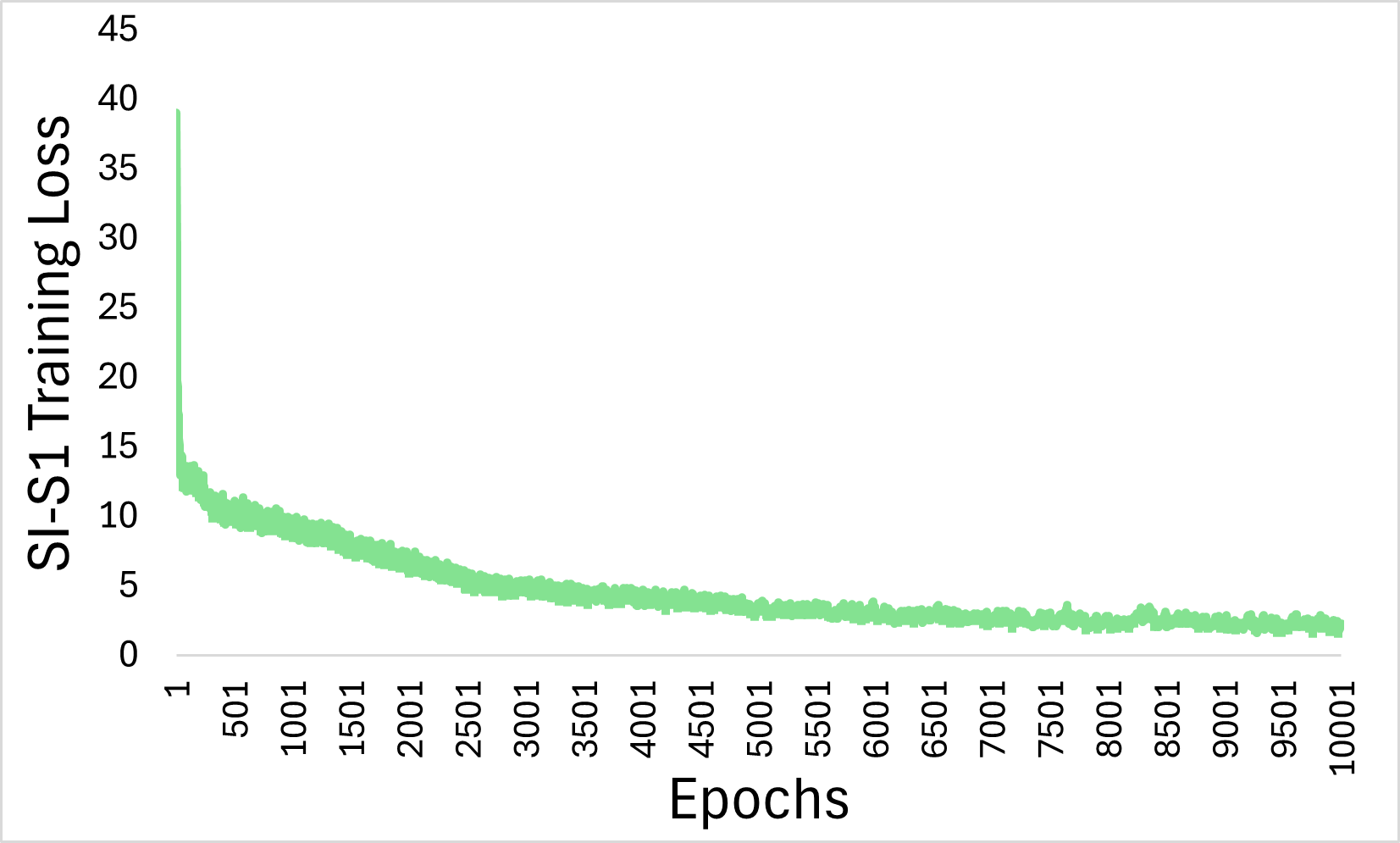

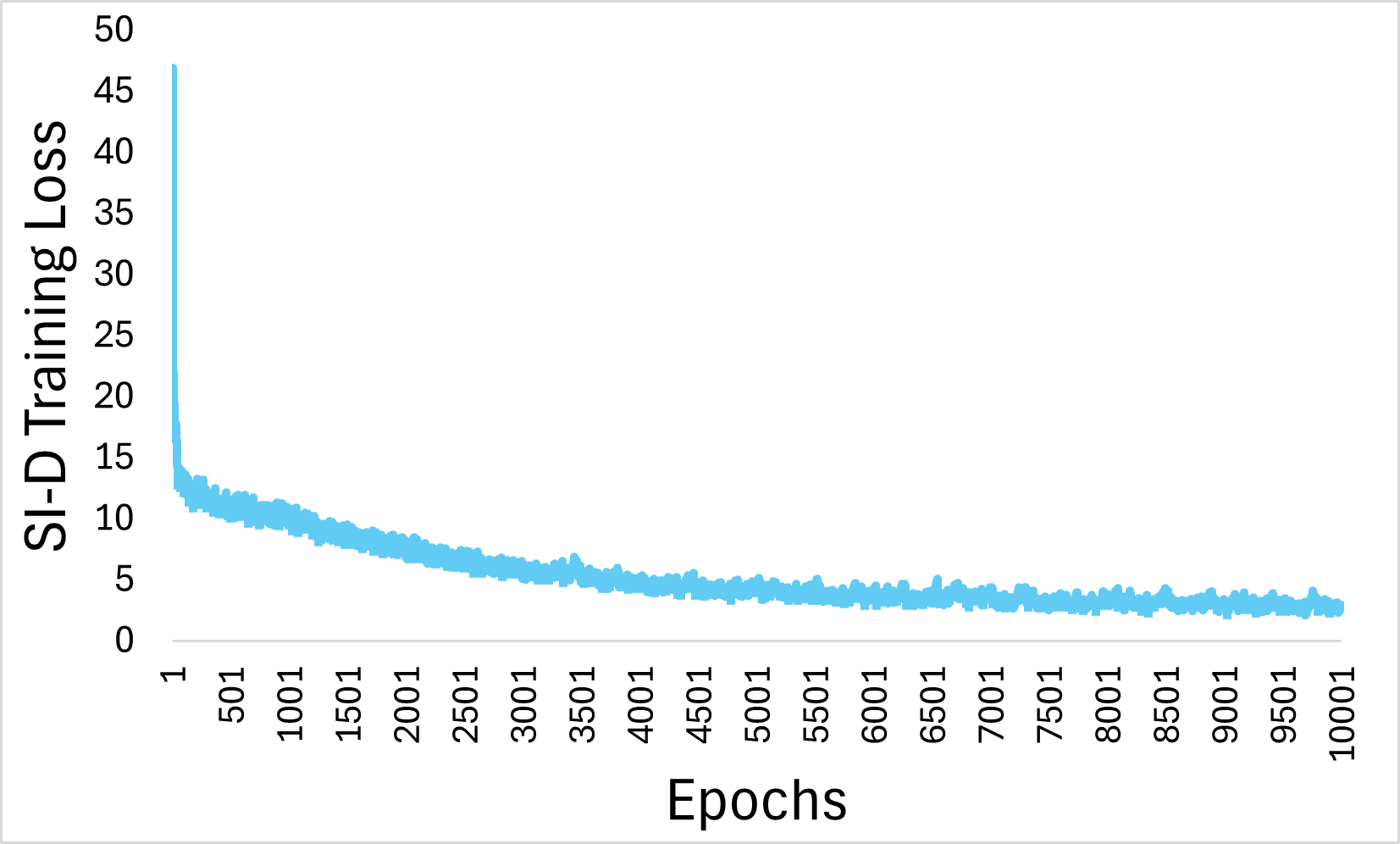


(a) SI-S1 Training Loss

(a) SI-S2 Training Loss

(a) SI-D Training Loss

Figure 3: Training loss convergence for SI-S1 and SI-S2 (Sepsis) and SI-D (Diabetes) policy learning.

# **References**

1. Argall BD, Chernova S, Veloso M, Browning B. A survey of robot learning from demonstration. Robotics and Autonomous Systems. 2009;57(5):469-83.
2. Castro PS, Li S, Zhang D. Inverse reinforcement learning with multiple ranked experts. arXiv preprint arXiv:190713411. 2019.
3. Ng AY, Russell SJ. Algorithms for inverse reinforcement learning. In: Proceedings of the 17th International Conference on Machine Learning; 2000. p. 663-70.
4. Armstrong S, Mindermann S. Occam’s razor is insufficient to infer the preferences of irrational agents. Advances in Neural Information Processing Systems. 2018;31.
5. Ho J, Ermon S. Generative adversarial imitation learning. In: Proceedings of the 30th International Conference on Neural Information Processing Systems; 2016. p. 4572-80.
6. Peng X, Ding Y, Wihl D, Gottesman O, Komorowski M, Lehman LwH, et al. Improv- ing sepsis treatment strategies by combining deep and kernel-based reinforcement learning. In: AMIA Annual Symposium Proceedings. vol. 2018; 2018. p. 887.
7. Raghu A, Komorowski M, Ahmed I, Celi L, Szolovits P, Ghassemi M. Deep rein- forcement learning for sepsis treatment. arXiv preprint arXiv:171109602. 2017.
8. Raghu A, Komorowski M, Celi LA, Szolovits P, Ghassemi M. Continuous state-space models for optimal sepsis treatment: A deep reinforcement learning approach. In: Machine Learning for Healthcare Conference. PMLR; 2017. p. 147-63.
9. Liu Z, Ji L, Jiang X, Zhao W, Liao X, Zhao T, et al. A deep reinforcement learn- ing approach for type 2 diabetes mellitus treatment. In: 2020 IEEE International Conference on Healthcare Informatics (ICHI). IEEE; 2020. p. 1-9.
10. Wang Z, Schaul T, Hessel M, Hasselt H, Lanctot M, Freitas N. Dueling network ar- chitectures for deep reinforcement learning. In: International Conference on Machine Learning. PMLR; 2016. p. 1995-2003.
11. Liu V, Escobar GJ, Greene JD, Soule J, Whippy A, Angus DC, et al. Hospital deaths in patients with sepsis from 2 independent cohorts. Jama. 2014;312(1):90-2.
12. Gotts JE, Matthay MA. Sepsis: Pathophysiology and clinical management. Bmj. 2016;353.
13. Marik PE. The demise of early goal-directed therapy for severe sepsis and septic shock. Acta anaesthesiologica Scandinavica. 2015;59(5):561-7.
14. Organization WH. Global report on diabetes [Internet]. 2016. Disponible sur: [http://apps](http://apps/) who int/iris/bitstream/10665/204871/1/9789241565257 eng pdf. 2017.
15. Singer M, Deutschman CS, Seymour CW, Shankar-Hari M, Annane D, Bauer M, et al. The third international consensus definitions for sepsis and septic shock (Sepsis- 3). Jama. 2016;315(8):801-10.
16. Javad MOM, Agboola SO, Jethwani K, Zeid A, Kamarthi S, et al. A reinforcement learning–based method for management of type 1 diabetes: Exploratory study. JMIR diabetes. 2019;4(3):e12905.
17. Oh SH, Park J, Lee SJ, Kang S, Mo J. Reinforcement learning-based expanded per- sonalized diabetes treatment recommendation using South Korean electronic health records. Expert Systems with Applications. 2022;206:117932.
